# LPE-Grown Lanthanide-MOF/Cellulose Paper for Visual Sensing and Selective Dye Removal

**DOI:** 10.3390/polym18172178

**Published:** 2026-09-07

**Authors:** Xiang Hou, Yipan Zeng, Yuhang Zhang, Yujie Li, Qutong Zheng

**Affiliations:** Research Center for Precision Medication of Chinese Medicine, College of Pharmacy, Hunan University of Chinese Medicine, Changsha 410208, China; 17373525352@163.com (X.H.); 18873940120@163.com (Y.Z.); 18573933076@163.com (Y.Z.); 17379937758@163.com (Y.L.)

**Keywords:** cellulose composite, liquid phase epitaxy, fluorescent paper, visual sensing, cationic dye adsorption

## Abstract

Cellulose-based functional materials have attracted increasing attention for portable environmental monitoring and pollutant management; however, achieving a robust integration of functional components with cellulose substrates remains challenging due to weak interfacial adhesion and instability of conventional coating strategies. Herein, a binder-free liquid-phase epitaxy (LPE) strategy was developed to construct lanthanide metal–organic framework (Ln-MOF) coatings directly on unmodified cellulose fibers, yielding a stable and multifunctional Ln-MOF/cellulose composite material. The LPE process enabled uniform growth of Ln-MOF layers on cellulose paper, resulting in homogeneous luminescence with relative standard deviations below 2% and stable fluorescence performance over a wide pH range of 3–11. By regulating the Eu^3+^/Tb^3+^ ratio, the obtained composite paper exhibited tunable dual-emission characteristics and enabled smartphone-assisted ratiometric visualization of dipicolinic acid (DPA), a representative biomarker of bacterial spores, with a linear response range of 0–2000 μM and a detection limit of 10 μM. Furthermore, the anionic Ln-MOF coating endowed the cellulose material with charge-selective adsorption capability, allowing efficient removal of cationic dyes while maintaining structural integrity after four regeneration cycles. The applicability of the LPE strategy was further demonstrated using different lanthanide–organic linker systems. This work provides a versatile approach for fabricating stable cellulose/MOF composite materials and highlights their potential applications in portable chemical sensing and selective water purification.

## 1. Introduction

Cellulose paper, a renewable polysaccharide-based material, combines flexibility, low density, capillary transport, processability, and low cost, making it an attractive substrate for portable analytical and separation platforms, including point-of-care testing (POCT) [1]. When integrated with luminescent components, paper-based materials enable rapid optical readout with minimal instrumentation and are therefore suitable for on-site analysis [2,3]. However, the performance of fluorescent paper depends not only on the intrinsic activity of the luminophore but also on the integrity of the interface between the functional phase and the cellulose fibers. Conventional fabrication methods, including impregnation, coating, and polymer-assisted immobilization, generally rely on physical adsorption or binder-mediated adhesion. Consequently, active particles may aggregate, detach, or leach, resulting in nonuniform loading, signal drift, and reduced operational stability. Polymer binders may additionally cover active sites, obstruct pores, and retard analyte transport [1,2,4,5,6,7,8]. Establishing a firmly anchored yet readily accessible functional coating on the fibrous cellulose network therefore remains a central challenge in the development of reliable paper-based polymer composites. The interfacial behavior of cellulose/MOF composites may depend on the cellulose source and the presence of residual non-cellulosic components such as hemicellulose and lignin. In the present study, a commercial qualitative cellulose filter paper was used as received; therefore, the conclusions of this work are restricted to this cellulose substrate rather than to cellulose materials of arbitrary composition.

Lanthanide metal–organic frameworks (Ln-MOFs) are promising functional components for addressing this challenge because they combine analyte-responsive luminescence with an ordered porous framework. Through the ligand-to-Ln^3+^ antenna effect, Ln-MOFs exhibit characteristic lanthanide emissions and energy-transfer pathways that can be modulated by guest molecules or coordination interactions [9,10]. In mixed-lanthanide systems, regulation of the relative Eu^3+^ and Tb^3+^ emissions enables dual-emission ratiometric sensing based on intensity ratios rather than absolute fluorescence intensity [11,12]. Such self-referenced signals can reduce the effects of uneven probe loading, excitation fluctuations, and background interference. The tunable coordination environments of Ln-MOFs can also produce ion-dependent photophysical responses, making them suitable for fluorescence-based discrimination of different ionic species. Furthermore, unlike conventional molecular dyes, Ln-MOFs provide both optical signal transduction and accessible porous structures. In particular, anionic Ln-MOF frameworks can electrostatically capture cationic guest molecules, providing an additional basis for charge-selective adsorption and separation [13,14]. Ln-MOF/cellulose composites may therefore integrate optical sensing and pollutant removal within a single flexible material.

Despite these advantages, most reported Ln-MOF/cellulose papers have been fabricated by impregnation, printing, or coating pre-synthesized MOF particles onto paper substrates [1,12,15,16,17,18]. In impregnation-based methods, MOF particles are mainly retained through physical interactions with cellulose fibers, which may result in particle aggregation, heterogeneous distribution, and leaching during use [19]. Printing and coating can improve macroscopic film formation but commonly require polymeric binders that may partially block MOF pores and limit the transport of target molecules [20]. These post-deposition approaches therefore face an inherent trade-off between interfacial adhesion and accessibility of the functional framework. Liquid-phase epitaxy (LPE), based on the sequential exposure of a substrate to metal-ion and organic-linker solutions, offers an alternative route for controlled, layer-by-layer MOF growth [21]. Rather than depositing preformed particles, LPE enables MOF nucleation and growth directly at the substrate interface and may thereby improve coating coverage and interfacial integration [22]. However, the direct use of LPE to construct binder-free Ln-MOF coatings on unmodified cellulose paper remains largely unexplored, particularly for multifunctional composites that combine fluorescence sensing and selective adsorption.

**Scheme 1:**
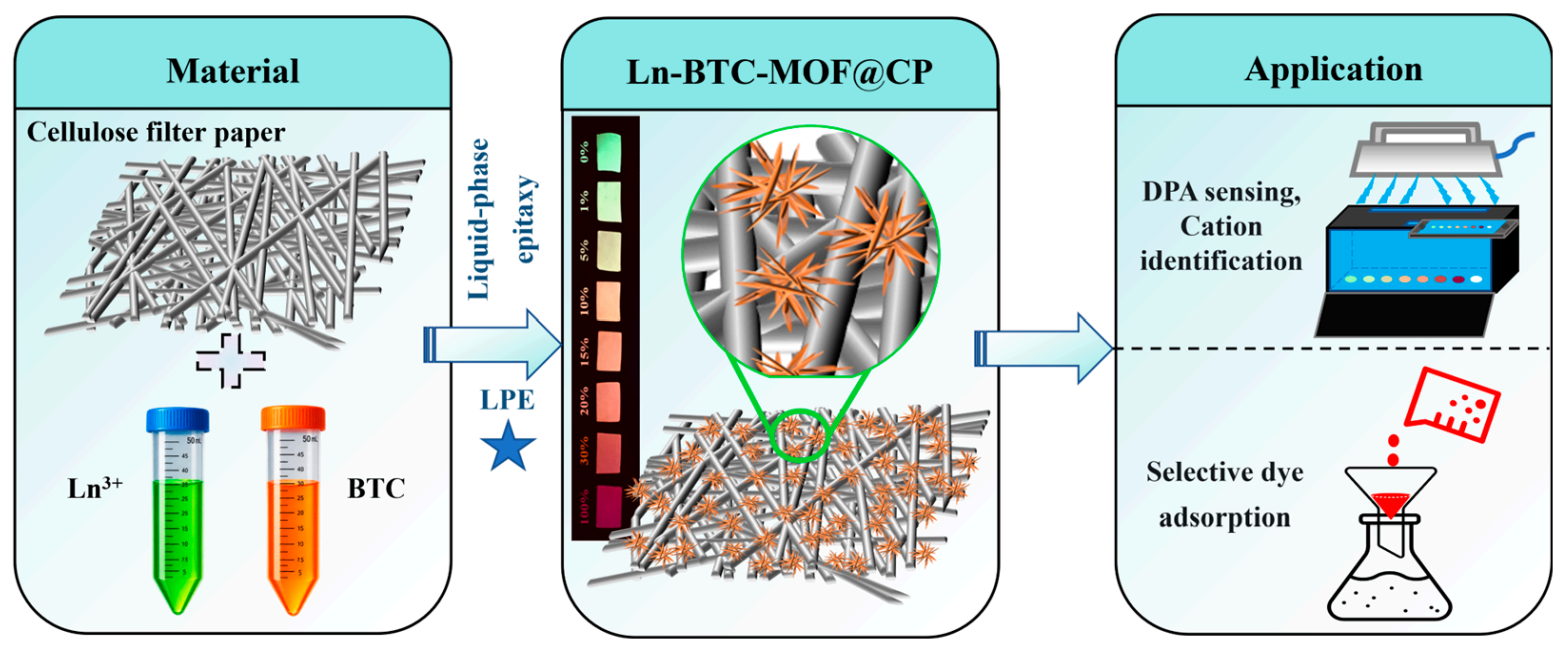
Ln-MOF fluorescent paper based on liquid phase epitaxy method is used for visual sensing of DPA, identification of cations, and selective removal of dyes. (The middle-figure inset shows Eu_x_Tb_1−x_-MOF@CP (x = 1, 0.3, 0.2, 0.15, 0.1, 0.05, 0.01, and 0) as imaged under a 254 nm UV lamp).

In this work, we report a binder-free LPE strategy for the in situ growth of Ln-MOF coatings on unmodified cellulose filter paper (Scheme 1). Mixed Eu/Tb-BTC was selected as the principal functional coating because regulation of the Eu^3+^/Tb^3+^ emission balance provides a self-referenced optical response. The resulting composite paper was investigated to determine whether sequential interfacial growth could improve coating uniformity and luminescence stability while preserving the flexibility and accessibility of the cellulose substrate. An optimized Eu/Tb-BTC MOF/cellulose paper enabled smartphone-assisted ratiometric detection of dipicolinic acid (DPA), a biomarker of bacterial endospores, through changes in the green-to-red fluorescence intensity ratio. Ion-dependent RGB responses were further used to construct three-dimensional decoding maps for the visual discrimination of selected cations and anions. In addition, the anionic Ln-MOF coating selectively captured cationic dyes during gravity-driven filtration and retained its adsorption performance over four regeneration cycles. The applicability of the LPE strategy was also examined using Ln-MOFs containing different organic linkers. Overall, this work establishes a relationship between LPE processing, fiber/MOF interfacial integration, and multifunctional performance, providing a general approach to stable cellulose/MOF composite materials for portable sensing and selective pollutant removal.

## 2. Experimental Section

### 2.1. Preparation of Ln-BTC MOF Powder

The luminescent Eu_0.1_Tb_0.4_-BTC MOF powder was prepared according to a reported method with minor modification [23]. Tb(NO_3_)_3_·6H_2_O (36.2 mg, 0.08 mmol) and Eu(NO_3_)_3_·6H_2_O (8.9 mg, 0.02 mmol) were dissolved in 5 mL of deionized water to form solution A. H_3_BTC (22 mg, 0.1 mmol) was dissolved in 5 mL of ethanol to form solution B. Solution A was added dropwise to solution B under magnetic stirring at 1000 rpm at room temperature, and the mixture was stirred for 1 h after precipitation. The product was collected by centrifugation, washed three times with ethanol and water, and dried under vacuum at 45 °C for 12 h.

### 2.2. Preparation of Fluorescent Paper (Ln-MOF@CP)

**Figure 1:**
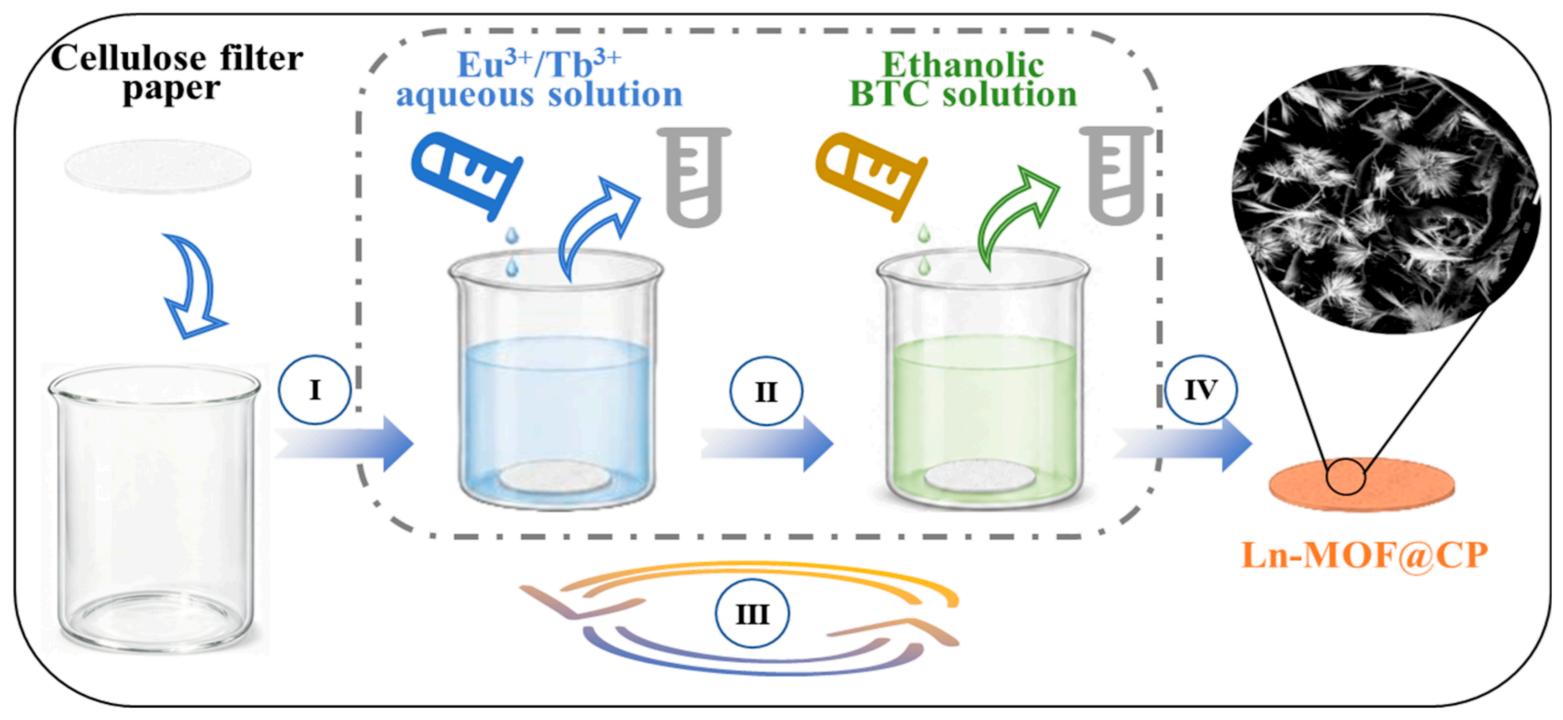
Schematic diagram of the preparation process of Ln-MOF@CP (CP: cellulose paper) by the liquid phase epitaxy method. (I: Adding an aqueous solution of metal ions; II: Adding an ethanol solution of the ligand; III: Repeating the process 8 times; IV: Washing/Drying).

Optimization of LPE cycles (8 cycles) and metal/ligand ratios is described in Supplementary Materials Section S3. The optimized condition used eight LPE cycles, which provided continuous MOF coverage while avoiding excessive crystal aggregation. For Eu_0.1_Tb_0.4_-MOF@CP, CP was immersed in 50 mL of an aqueous solution containing Eu(NO_3_)_3_·6H_2_O (44.6 mg, 0.1 mmol) and Tb(NO_3_)_3_·6H_2_O (181 mg, 0.4 mmol) for 5 min, then transferred into 50 mL of an ethanolic BTC solution (0.5 mmol) for another 5 min. This metal-solution/ligand-solution sequence was repeated eight times. We rinsed the obtained paper three times with water and ethanol and dried under vacuum at 45 °C for 12 h (Figure 1). Eu_x_Tb_1−x_-MOF@CP samples (x = 1, 0.3, 0.2, 0.15, 0.1, 0.05, 0.01, and 0) were prepared by adjusting the Eu/Tb precursor ratio under the same LPE protocol.

### 2.3. Visual Identification of Ions

Dried Eu_0.1_Tb_0.4_-MOF@CP was cut into circular discs (6 mm diameter) using a hole punch. Each disc was immersed in 500 μL of aqueous ion solution for 5 min and then photographed under a 254 nm UV lamp using a smartphone in a fixed imaging setup. Unless otherwise stated, each measurement was repeated at least three times. RGB values were extracted using the World of Color app (Maarten Zonneveld) from an identical circular region of interest. A water-treated Eu_0.1_Tb_0.4_-MOF@CP disc was used as the blank. The normalized chromaticity ratios R/R_0_, B/B_0_, and G/G_0_ were used as the X-, Y-, and Z-coordinates, respectively, where R_0_, B_0_, and G_0_ are the blank RGB values. These normalized coordinates were used to construct three-dimensional decoding maps for ion discrimination.

### 2.4. Visual Fluorescent Sensing of DPA

Dried Eu_0.1_Tb_0.4_-MOF@CP was cut into 6 mm discs and used as a paper-based visual sensor. The discs were immersed in 500 μL of DPA aqueous solutions (0–2000 μM) for 5 min and then analyzed using a self-designed visual detection device with 254 nm excitation. Fluorescent images were recorded by smartphone under fixed conditions. RGB values were extracted using the World of Color app, and the DPA concentration was evaluated from the green/red chromaticity ratio (G/R).

### 2.5. Selective Removal of Cationic Dyes

For dye adsorption, CP or Eu_0.1_Tb_0.4_-MOF@CP was folded into a conical glass funnel, and a vial was placed below the funnel to collect the filtrate. Aqueous dye solutions (4 mL, 5 ppm) were prepared using MO^−^, MB^+^, IC^−^, R_6_G^+^, SD_0_, or mixed dyes. The solutions passed through the paper by gravity filtration, and the filtrates were analyzed by UV-vis spectroscopy. For regeneration, dye-loaded Eu_0.1_Tb_0.4_-MOF@CP was alternately washed three times with saturated NaCl solution in DMF and methanol. The exact NaCl-DMF composition should be completed in the final experimental record. The regenerated paper was dried under vacuum at 45 °C for 12 h before the next adsorption cycle. Four adsorption regeneration cycles were performed.

### 2.6. Calculation Methods

Density functional theory calculations were performed using Gaussian 09 to analyze the electronic structure of BTC. The ground-state electronic structure of BTC was optimized and calculated at the B_3_LYP/6-31G* level. Excited-state calculations were performed using the TD-SCF/B_3_LYP/6-31G* method.

## 3. Results and Discussion

### 3.1. Optimization of Preparation Conditions

To obtain fluorescent paper with a uniform coating and stable fluorescence, we optimized the preparation conditions using Tb(BTC)(H_2_O)_6_ as the fluorescent coating model. The detailed preparation parameters are listed in Table S1. Methods 1, 2, 3, 4, and 5 were based on in-situ growth under different conditions, while Methods 6, 7, 8, and 9 used liquid-phase epitaxial layer-by-layer growth with varied parameters.

The growth of the Tb-BTC MOF coating on cellulose paper was first examined under a 254-nm UV lamp. As shown in Figure S1a, Tb-MOF@CP prepared by in-situ growth, including Methods 1, 2, 3, 4, and 5 showed uneven green fluorescence. In contrast, the samples prepared by liquid-phase epitaxy exhibited much more uniform fluorescence. This difference is mainly due to the different crystal growth behaviors. In-situ growth provides poor control over crystal orientation, making it difficult to form an ordered and uniform coating. By contrast, liquid-phase epitaxy promotes directional layer-by-layer growth of Tb-BTC MOF crystals, leading to a highly oriented and uniformly dense Tb-MOF@CP coating, consistent with previous reports [24].

To further compare the LPE conditions used in Methods 6, 7, 8, and 9 the resulting Tb-MOF coatings were characterized by scanning electron microscopy (SEM) and powder X-ray diffraction (PXRD).

#### 3.1.1. Analysis of PXRD Results

The PXRD patterns of Tb-MOF@CP prepared by the four methods are shown in Figure S1b. Methods 7 and 9 gave relatively weak diffraction peaks, whereas Methods 6 and 8 showed slightly stronger peaks, suggesting a higher amount of Tb-BTC MOF crystals on the cellulose surface. All four samples displayed characteristic peaks of both cellulose paper (CP) and Tb-BTC MOF, confirming the successful formation of Tb-BTC MOF-coated fluorescent paper, in agreement with the literature [25].

#### 3.1.2. Analysis of SEM Results

SEM images in Figure S2 show that bundled crystals formed on the surface of Tb-MOF@CP prepared by all four methods, but their size and distribution differed markedly. Methods 7 and 9 produced relatively sparse surface crystals, while Methods 6 and 8 gave dense, uniform, and similarly sized crystals. This suggests that the washing step during LPE cycling may be omitted, as washing may remove Tb^3+^ ions or ligands adsorbed on cellulose and interfere with the layer-by-layer growth of Tb-BTC MOF. This effect can be partly compensated by increasing the number of coating cycles.

#### 3.1.3. Selection of Preparation Method

Compared with Method 6, Method 8 produced a more regular and uniform crystal structure on Tb-MOF@CP. This result indicates that the additives used during preparation strongly affect the crystallinity of the luminescent coating, as also reported previously [26,27]. Based on these results, Method 8 was selected for preparing fluorescent paper with an Ln-MOF fluorescent coating.

To obtain stronger luminescence, the number of epitaxial growth cycles was further optimized according to the literature [28].

#### 3.1.4. Analysis of Fluorescence Spectra and Intensity

Figure S3 shows the fluorescence spectra and intensity changes of Tb-MOF@CP prepared with different cycle numbers. The fluorescence intensity increased gradually with the number of cycles, but changed little after 8 cycles. The inset in Figure S3a shows the fluorescence images of the corresponding samples under a 254-nm UV lamp, further supporting this trend.

#### 3.1.5. Analysis of SEM Results

SEM images in Figure S4 further show the effect of cycle number on MOF coating growth. As the number of cycles increased, the fluorescent MOF coating on cellulose paper (CP) became denser. After 8 cycles, the CP surface was fully covered by densely grown MOF crystals. Further increasing the cycle number mainly increased the coating layers, with little improvement in fluorescence performance.

#### 3.1.6. Selection of the Number of Cycles

Based on the fluorescence spectra, fluorescence intensity, and SEM results, 8 cycles was selected as the optimal number for preparing fluorescent paper by the liquid-phase epitaxial method.

### 3.2. Homogeneity and Stability

In practical use, the fluorescence uniformity and stability of fluorescent paper are key factors for reliable detection, as they help reduce false-positive or false-negative results [29]. Therefore, the fluorescence uniformity and stability of the prepared Tb-BTC MOF@CP were evaluated.

#### 3.2.1. Study on Fluorescence Uniformity

Fluorescence uniformity was assessed by measuring emission intensities at different positions on the same fluorescent paper and among different batches. As shown in Figure S5, the fluorescence intensities remained nearly consistent in both cases, with relative standard deviations (RSD, n = 3) of 1.82% and 0.93%, respectively. These low RSD values confirm the good fluorescence uniformity of Tb-BTC MOF@CP prepared by this method.

#### 3.2.2. Study on Fluorescence Stability

Fluorescence stability was evaluated by monitoring the Tb^3+^ emission at 546 nm (^5^D_4_ → ^7^F_5_) over time. As shown in Figure S6, the fluorescence intensity of the Tb-BTC MOF aqueous dispersion decreased by about 60% after 30 min, mainly due to particle aggregation and sedimentation in water, as shown in the inset of Figure S6a and reported previously [29]. The soaked Tb-BTC MOF@CP showed a smaller but still obvious fluorescence decrease of about 15% after 30 min, likely because MOF particles were only weakly adsorbed on the cellulose surface. In contrast, the LPE-prepared Tb-BTC MOF@CP maintained nearly unchanged fluorescence intensity within 30 min, demonstrating superior fluorescence stability. Overall, the liquid-phase epitaxial method provides fluorescent paper with improved uniformity and stability, supporting its potential for practical applications.

### 3.3. Physical Characterization

**Figure 2:**
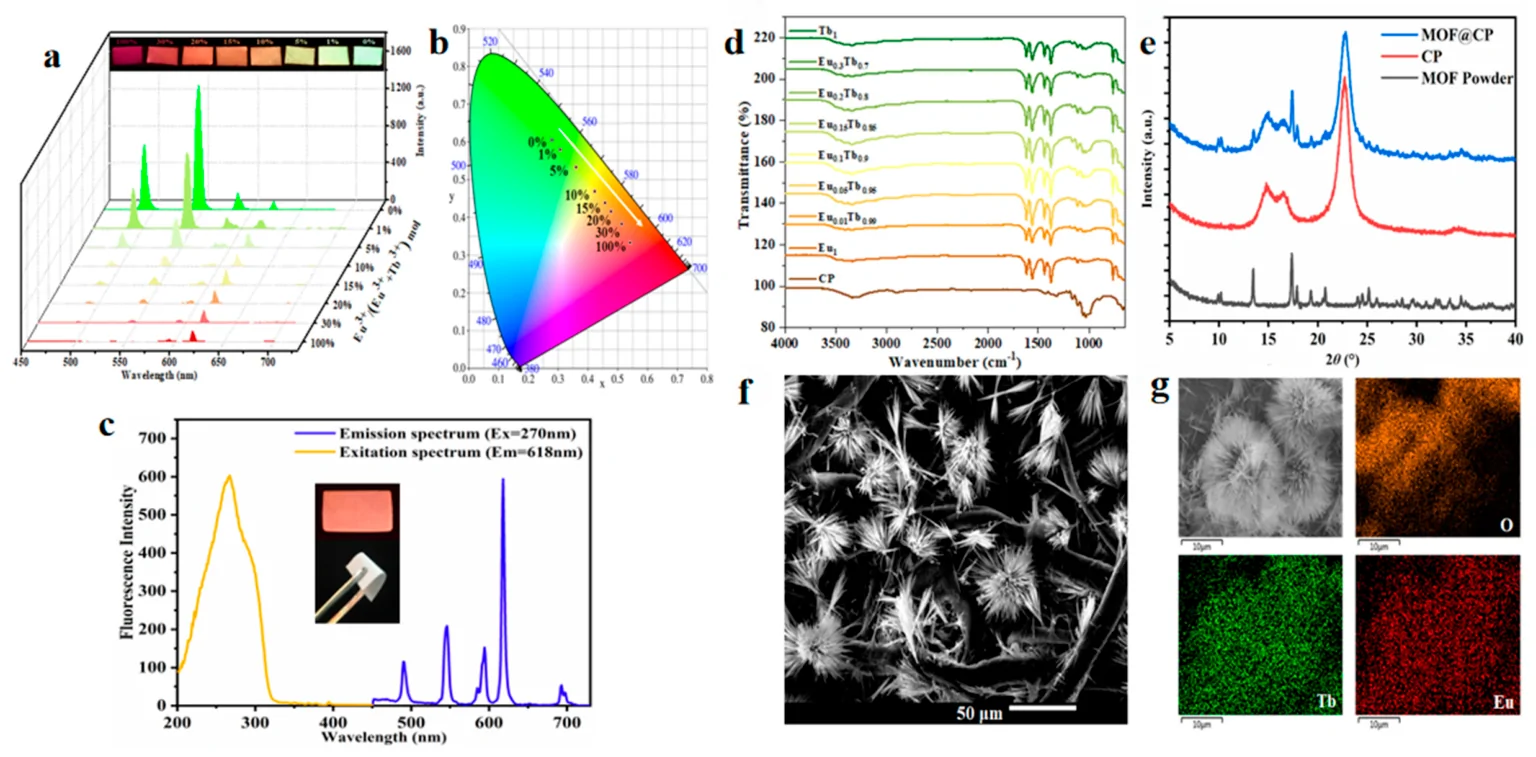
(**a**) Emission spectra of Eu_x_Tb_1−x_-MOF@CP (x = 1, 0.3, 0.2, 0.15, 0.1, 0.05, 0.01 and 0) in an aqueous solution (λ_ex_ = 278 nm), and the inset is the corresponding image under a 254 nm ultraviolet lamp; (**b**) CIE chromaticity diagram of Eu_x_Tb_1−x_-MOF@CP (x = 1, 0.3, 0.2, 0.15, 0.1, 0.05, 0.01 and 0); (**c**) Excitation and emission spectra of Eu_0.1_Tb_0.4_-MOF@CP. The insets show the fluorescence image (λ_ex_ = 254 nm) and the folded macroscopic view of Eu_0.1_Tb_0.4_-MOF@CP; (**d**) Infrared absorption spectra of Eu_x_Tb_1−x_-MOF@CP (x = 1, 0.3, 0.2, 0.15, 0.1, 0.05, 0.01 and 0); (**e**) PXRD patterns of Eu_0.1_Tb_0.4_−BTC MOF powder, CP and Eu_0.1_Tb_0.4_-MOF@CP; (**f**) SEM image of Eu_0.1_Tb_0.4_-MOF@CP; (**g**) EDS image of Eu_0.1_Tb_0.4_-MOF@CP.

A series of Eu_x_Tb_1−x_-MOF@CP samples (x = 1, 0.3, 0.2, 0.15, 0.1, 0.05, 0.01, and 0) were prepared because different lanthanide ions have similar coordination behavior and can be co-introduced into the MOF lattice [18]. FT-IR spectra (Figure 2d) showed similar chemical-bond compositions for all samples. The absorption bands at 1450–1370 cm^−1^ and 1615–1550 cm^−1^ were assigned to symmetric and asymmetric stretching vibrations of carboxylate groups in BTC [30]. Therefore, Eu_0.1_Tb_0.4_-MOF@CP was selected for subsequent performance evaluation.

PXRD and SEM confirmed the crystalline, uniformly distributed Eu_0.1_Tb_0.4_-BTC MOF layer on cellulose fibers (Figure 2e,f). EDS elemental mapping further confirmed homogeneous Eu and Tb distribution across the cellulose fibers (Figure 2g). The Brunauer–Emmett–Teller (BET) specific surface area of Eu_0.1_Tb_0.4_-MOF@CP was measured to be 1.97 m^2^/g, with an average pore diameter of 21.86 nm (Figure S6d). The mesoporous structure facilitates rapid diffusion and accessibility of target analytes, which is favorable for both sensing response and dye adsorption kinetics.

### 3.4. Luminescent Properties

All Ln-MOF@CP samples exhibit characteristic emissions with narrow, well-separated bands. To understand the energy transfer within Eu_x_Tb_1−x_-MOF@CP, we plotted the ligand’s excitation energy level diagram (Figure S7b) and calculated its singlet and triplet states using density functional theory (DFT). The BTC energy gap △EST (1ππ*–3ππ*) is 8221 cm^−1^ (>5000 cm^−1^), indicating efficient intersystem crossing (ISC) and effective sensitization of both Eu^3+^ and Tb^3+^ emissions [31,32].

The fluorescent colors of bimetallic Ln-MOF@CP can be tuned by adjusting the Eu^3+^/Tb^3+^ ratio. Figure S8 show the emission spectra and CIE chromaticity coordinates for Eu_x_Tb_1−x_-MOF@CP (x = 1, 0.3, 0.2, 0.15, 0.1, 0.05, 0.01, and 0) in solid and aqueous states. Fluorescence intensity decreases in water due to O-H vibrational quenching, but the color remains unchanged [33]. BTC acts as an “antenna” chromophore, sensitizing both Eu^3+^ and Tb^3+^. Excitation at 278 nm produces tunable emission by modulating Eu^3+^ and Tb^3+^ intensities (Figure S8a and Figure 2a). Increasing Tb^3+^ content shifts the emission from red to green, visible to the naked eye (insets in Figure S8a), consistent with calculated CIE coordinates (Figure S8b and Figure 2b).

Eu_0.1_Tb_0.4_-MOF@CP was selected as a fluorescent sensor due to comparable Eu^3+^ and Tb^3+^ emission intensities [12]. Excitation at 270 nm produces Eu^3+^ peaks at 594 nm and 618 nm (^5^D_0_-^7^F_J_ J = 1, 2) and Tb^3+^ peaks at 490 nm and 546 nm (^5^D_4_-^7^F_J_ J = 5, 6). Despite higher Tb^3+^ content, Eu^3+^ emission dominates due to energy transfer from Tb^3+^ to Eu^3+^ [23], giving Eu_0.1_Tb_0.4_-MOF@CP its orange-red fluorescence (Figure S8a, upper inset). Fluorescence lifetimes are 361.4 μs (Tb^3+^, 546 nm) and 188.7 μs (Eu^3+^, 618 nm) (Figure S10), confirming efficient ligand-to-Ln^3+^ energy transfer via the “antenna effect” [34].

Eu_0.1_Tb_0.4_-MOF@CP retains the flexibility and cuttability of cellulose filter paper (Figure 2d, lower inset) and can be cut into 6 mm circular pieces for visual sensing (Figure S9a). Its fluorescence remains stable across a wide pH range (3–11), maintaining orange-red emission under 254 nm UV irradiation (Figure S9b, inset), indicating suitability for analyte detection in acidic and alkaline conditions. The absolute photoluminescence quantum yield of the composite paper was determined to be 11.4% under 278 nm excitation. This sufficient emission efficiency ensures bright and distinguishable fluorescence signals, enabling reliable naked-eye and smartphone-based visual detection without the need for complex instrumentation.

### 3.5. Visual Identification of Ions

Metal cations are relevant to pharmaceutical quality, food safety, and environmental monitoring, while organic anions play key roles in physiological and ecological systems [33,35,36]. Organic anions are also important in physiological processes and ecological systems [34]. Rapid, economical, and portable methods for identifying metal ions and anions are therefore highly desirable. Although fluorescence-based methods for distinguishing anions [37,38] and cations [39,40,41] have been reported, many rely on intensity-based readouts and costly, non-portable instruments. In contrast, digital image-based colorimetric fluorescence analysis offers a practical strategy for high-throughput on-site ion discrimination [36,42,43].

Different ions may perturb ligand-to-lanthanide energy transfer or alter the emission distribution between Tb^3+^ and Eu^3+^ centers to different extents [11], thereby inducing distinguishable fluorescence color changes. Accordingly, Eu_0.1_Tb_0.4_-MOF@CP was selected for cation and anion sensing based on RGB chromaticity values, owing to its strong luminescence and broad-pH aqueous stability.

Experimentally, a 6 mm Eu_0.1_Tb_0.4_-MOF@CP disc was immersed in 500 μL of 1 mM aqueous ion solution for 5 min. The treated disc was photographed under a 254 nm UV lamp using a smartphone, and its R, G, and B channel intensities were extracted using the World of Color application. A water-treated Eu_0.1_Tb_0.4_-MOF@CP disc served as the blank control for color calibration.

**Figure 3:**
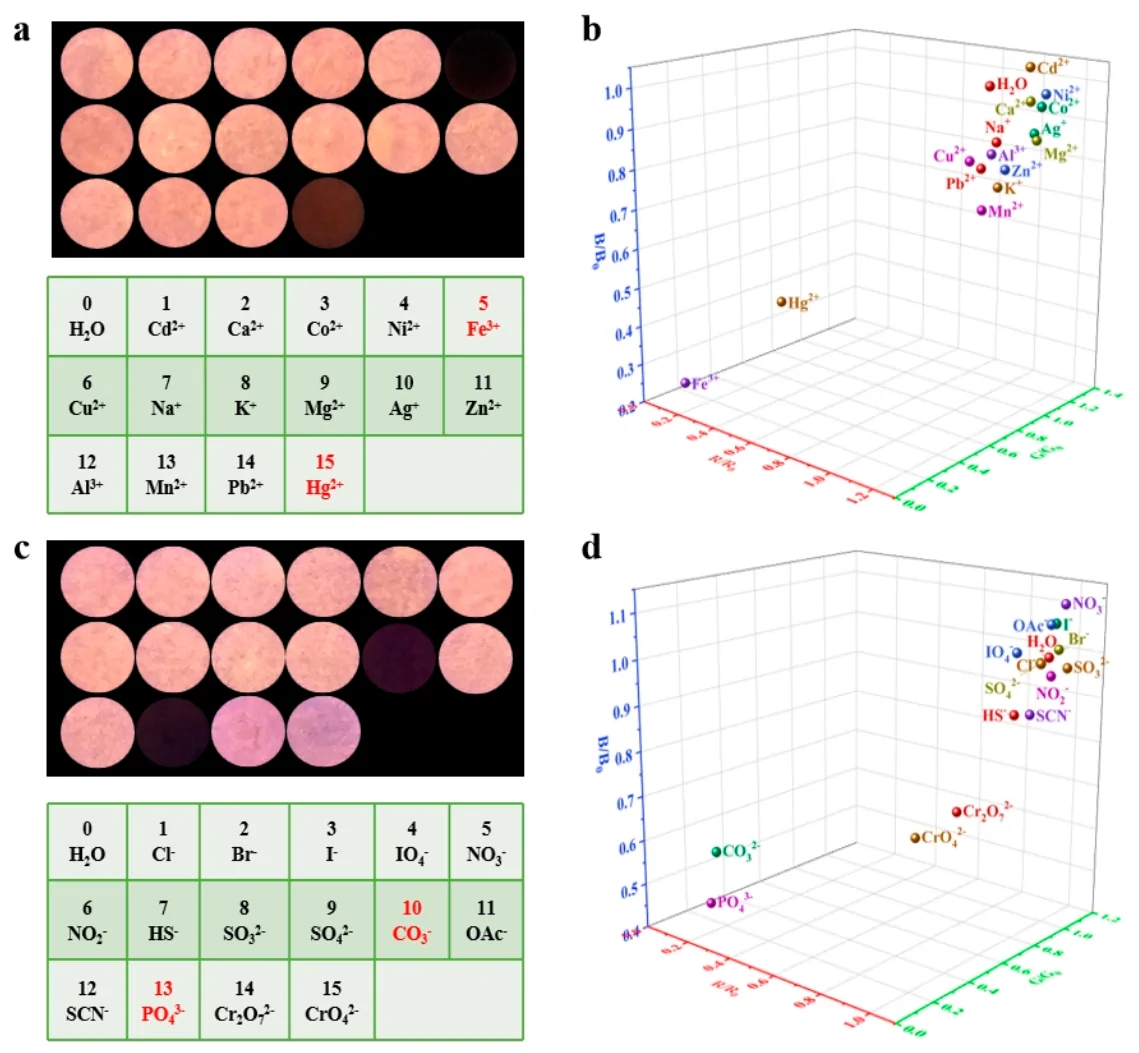
(**a**) Fluorescent color images of Eu_0.1_Tb_0.4_-MOF@CP in aqueous solutions of different metal cations (λ_ex_ = 254 nm); (**b**) Three-dimensional decoding map of metal cations with the chromaticity value intensity ratios R/R_0_, B/B_0_, and G/G_0_ as the x-, y-, and z-axes; (**c**) Fluorescent color images of Eu_0.1_Tb_0.4_-MOF@CP in aqueous solutions of different anions (λ_ex_ = 254 nm); (**d**) Three-dimensional decoding map of anions with the chromaticity value intensity ratios R/R_0_, B/B_0_, and G/G_0_ as the x-, y-, and z-axes.

As shown in Figure 3a,c, different ions produced different effects on the fluorescence color of Eu_0.1_Tb_0.4_-MOF@CP. In particular, Fe^3+^, CO_3_^2−^, and PO_4_^3−^ significantly quenched the orange-red fluorescence, whereas the other tested ions caused only slight changes in the characteristic emission color. Eu_0.1_Tb_0.4_-MOF@CP selectively identifies Fe^3+^, CO_3_^2−^, and PO_4_^3−^ in aqueous solution through distinct visual fluorescence changes.

To further distinguish the ion responses, three-dimensional decoding maps were constructed using the normalized RGB intensity ratios B/B_0_, R/R_0_, and G/G_0_, where B, R, and G are the chromaticity values after ion treatment, and B_0_, R_0_, and G_0_ are those of the blank control. In the 3D decoding maps, R/R_0_, B/B_0_, and G/G_0_ were assigned as the X, Y, and Z axes, respectively. As shown in Figure 3b,d, each tested cation or anion occupies a characteristic coordinate in the 3D space, enabling straightforward visual discrimination.

The RGB-coordinate distribution also provides useful insight into the sensing behavior. An increase in the X value reflects an enhanced contribution from red Eu^3+^ emission, which may be associated with more efficient ligand-to-Eu^3+^ sensitization or Tb^3+^-to-Eu^3+^ energy transfer. An increase in the Z value indicates a higher proportion of green Tb^3+^ emission, suggesting suppressed Eu^3+^ sensitization or weakened Tb^3+^-to-Eu^3+^ energy transfer. A pronounced deviation in the Y value may be related to changes in the blue-background component, scattering, or structural perturbation of the MOF coating. Therefore, the ion-dependent RGB response of Eu_0.1_Tb_0.4_-MOF@CP can be used to effectively distinguish different metal cations and anions in aqueous solution.

### 3.6. Visual Sensing of DPA

Bacillus anthracis is highly pathogenic, and rapid detection of its biomarker pyridine-2,6-dicarboxylic acid (DPA) is important for biosafety monitoring [44]. Lanthanide-based sensors have been developed for DPA detection, but many methods require complex operation or expensive equipment [45]. Therefore, Eu_0.1_Tb_0.4_-MOF@CP was evaluated as a portable, low-cost visual sensor for DPA.

**Figure 4:**
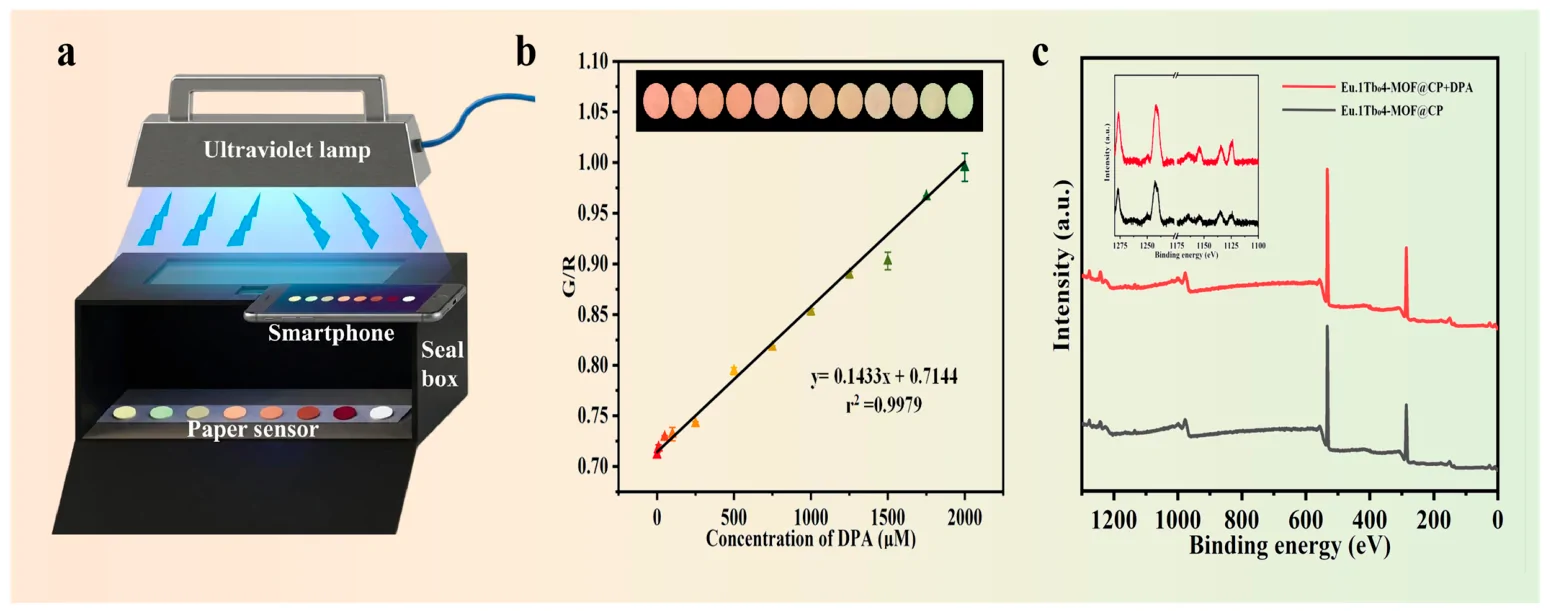
(**a**). Visual detection device for DPA; (**b**). Calibration curve and fluorescence images of the paper-based sensor after being immersed in DPA solutions of different concentrations (λ_ex_ = 254 nm); (**c**) XPS spectra of Eu_0.1_Tb_0.4_-MOF@CP before and after contact with DPA and XPS spectra of Tb and Eu.

For DPA sensing, Eu_0.1_Tb_0.4_-MOF@CP discs were immersed in 500 μL of DPA solutions (0, 10, 50, 100, 250, 500, 750, 1000, 1250, 1500, 1750, and 2000 μM) for 5 min. Under 254 nm UV irradiation, the fluorescence color changed from orange-red to green and could be distinguished by the naked eye (Figure 4b inset). Smartphone images were further analyzed to obtain R and G values, and the G/R ratio was used as the ratiometric signal. The G/R ratio showed a linear relationship with DPA concentration from 0 to 2000 μM with a correlation coefficient of 0.9979. The fitting equation was y = 1.433 × 10^−4^x + 0.7144. The limit of detection was 10 μM, lower than the reported minimum infectious dose level of anthrax spores to humans (60 μM) [46]. Although this LOD is higher than those of some instrument-based ratiometric fluorescence methods, the paper device provides simple operation, low cost, portability, and direct visual readout for on-site DPA screening. To evaluate the detection selectivity of the Eu_0.1_Tb_0.4_-MOF@CP paper-based sensor toward DPA, the fluorescence response of the sensor was investigated in the presence of various potential interfering substances. The results showed that under identical experimental conditions, other interfering substances only caused negligible changes in the relative G/R value of the sensor, while the addition of DPA induced a remarkable increase in this ratio. The corresponding fluorescence imaging results further verified that only DPA could trigger an obvious color transition of the sensing film from orange-red to green (Figure S16). This excellent selectivity can be attributed to the favorable binding geometry and synergistic chelation effect of the DPA moiety, which enables DPA to preferentially coordinate with the Tb^3+^ active sites.

### 3.7. Sensing Mechanism

It has been reported that metal cations or anions can quench Ln-MOF fluorescence through: (1) interaction with organic ligands; (2) collapse of the MOF crystal structure; and (3) ion exchange with central cations [47]. To explore this, Eu_0.1_Tb_0.4_-MOF@CP was soaked in aqueous solutions of Fe^3+^, CO_3_^2−^, or PO_4_^3−^ and analyzed by FT-IR and PXRD (Figure S11). Compared with untreated samples, the FT-IR spectra show significant chemical changes, while PXRD patterns indicate amorphization with disappearing diffraction peaks. These results suggest that Fe^3+^, CO_3_^2−^, or PO_4_^3−^ disrupt the Eu_0.1_Tb_0.4_-BTC MOF crystal framework, causing loss of orange-red luminescence.

It should be noted that this study provides a preliminary evaluation of Eu_0.1_Tb_0.4_-MOF@CP for sensing Fe^3+^, CO_3_^2−^, or PO_4_^3−^. Considering the importance of both sensing performance and material stability, further research is required to assess the practical feasibility of this fluorescent paper in ion detection applications.

**Figure 5:**
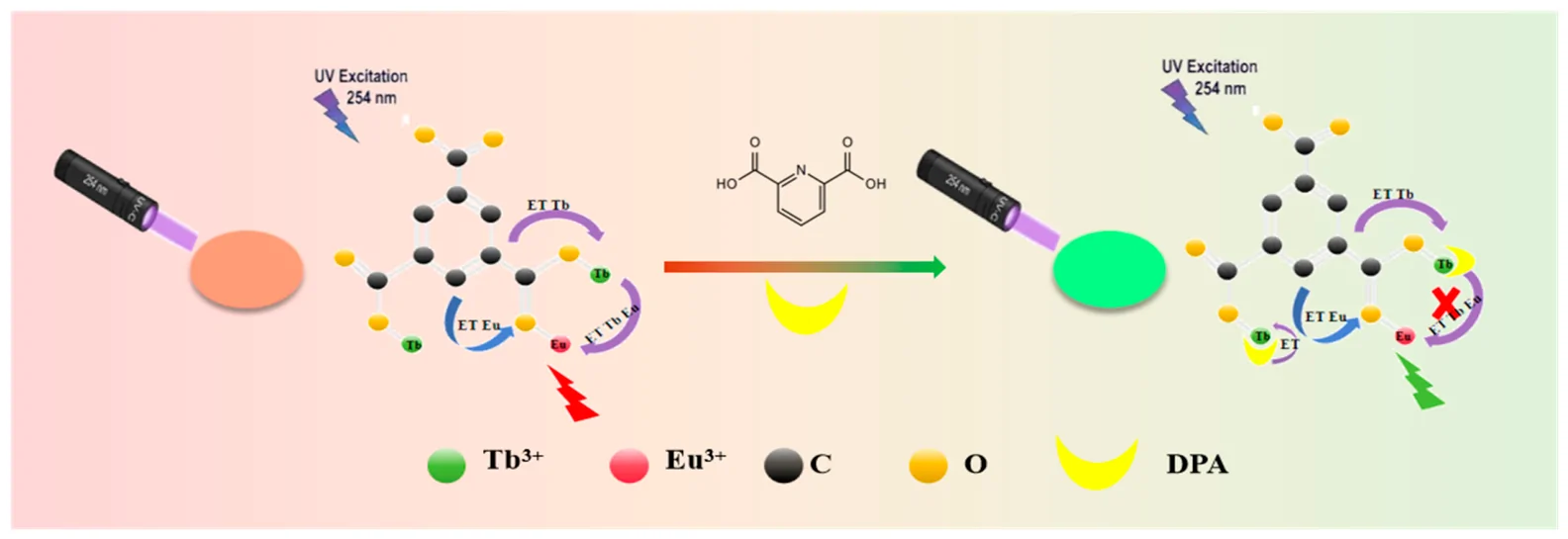
Schematic diagram of the sensing mechanism of Eu_0.1_Tb_0.4_-MOF@CP for DPA.

The visual sensing mechanism of Eu_0.1_Tb_0.4_-MOF@CP toward DPA is illustrated in Figure 5. Upon the addition of DPA, the fluorescence color of Eu_0.1_Tb_0.4_-MOF@CP changes from orange-red to green, which can be attributed to the redistribution of the energy-transfer pathways within the lanthanide MOF system [12].

Before exposure to DPA, the luminescence of Eu_0.1_Tb_0.4_-MOF@CP mainly originates from two energy-transfer processes. The first is the antenna effect from the 1,3,5-benzenetricarboxylic acid (BTC) ligand to Ln^3+^ centers, and the second is the intermetallic energy transfer from Tb^3+^ to Eu^3+^. As a result, the material exhibits orange-red emission under 254 nm excitation. After DPA is introduced, DPA molecules coordinate preferentially with Tb^3+^ sites to form Tb-DPA complexes, thereby altering the original energy-transfer process.

DPA coordinates preferentially with Tb^3+^, as evidenced by the XPS shift (Figure 4c). After treatment with DPA, the Tb 3d_5/2_ peak of Eu_0.1_Tb_0.4_-MOF@CP shifts from 1242.3 eV to a lower binding-energy region at 1241.6 eV, whereas the Eu 4d signal shows negligible change. This result suggests a stronger interaction between DPA and Tb^3+^ than between DPA and Eu^3+^. The decrease in the Tb 3d_5/2_ binding energy can be attributed to the formation of Tb-O and Tb-N coordination bonds, which increases the electron density around Tb^3+^.

Because DPA possesses strong ultraviolet absorption, it can act as an efficient antenna ligand after coordination with Tb^3+^, promoting energy transfer from DPA to Tb^3+^. Meanwhile, the coordinated DPA molecules disturb the original Ln^3+^-centered energy-transfer process, particularly the Tb^3+^-to-Eu^3+^ energy transfer [34,48]. Consequently, the Tb^3+^-centered green emission is enhanced, while the Eu^3+^-centered red emission is relatively suppressed, leading to an obvious fluorescence color change from orange-red to green.

Therefore, the visual response of Eu_0.1_Tb_0.4_-MOF@CP toward DPA can be mainly ascribed to two factors: first, DPA rapidly coordinates with Tb^3+^ to form Tb-DPA complexes and sensitizes Tb^3+^ emission; second, DPA coordination interferes with the Tb^3+^-to-Eu^3+^ energy-transfer pathway. These two effects jointly contribute to the ratiometric fluorescence response and enable visual detection of DPA by Eu_0.1_Tb_0.4_-MOF@CP.

### 3.8. Selective Removal of Dyes

Ln-BTC MOFs possess a suitable pore volume of approximately 0.23 cm^3^/g and an anionic framework [13,49], enabling charge-selective adsorption of guest molecules. On this basis, Eu_0.1_Tb_0.4_-MOF@CP was evaluated for selective removal of organic dyes with different charges.

The dye adsorption process is simple and does not require additional equipment. Eu_0.1_Tb_0.4_-MOF@CP was folded in a glass funnel, and dye solution was passed through the paper. The filtrate was collected and analyzed by UV–vis spectroscopy. Methylene blue (MB^+^) and methyl orange (MO^−^), with comparable molecular sizes but opposite charges, were selected as model dyes. For comparison, CP and Eu_0.1_Tb_0.4_-MOF@CP were used as filters.

**Figure 6:**
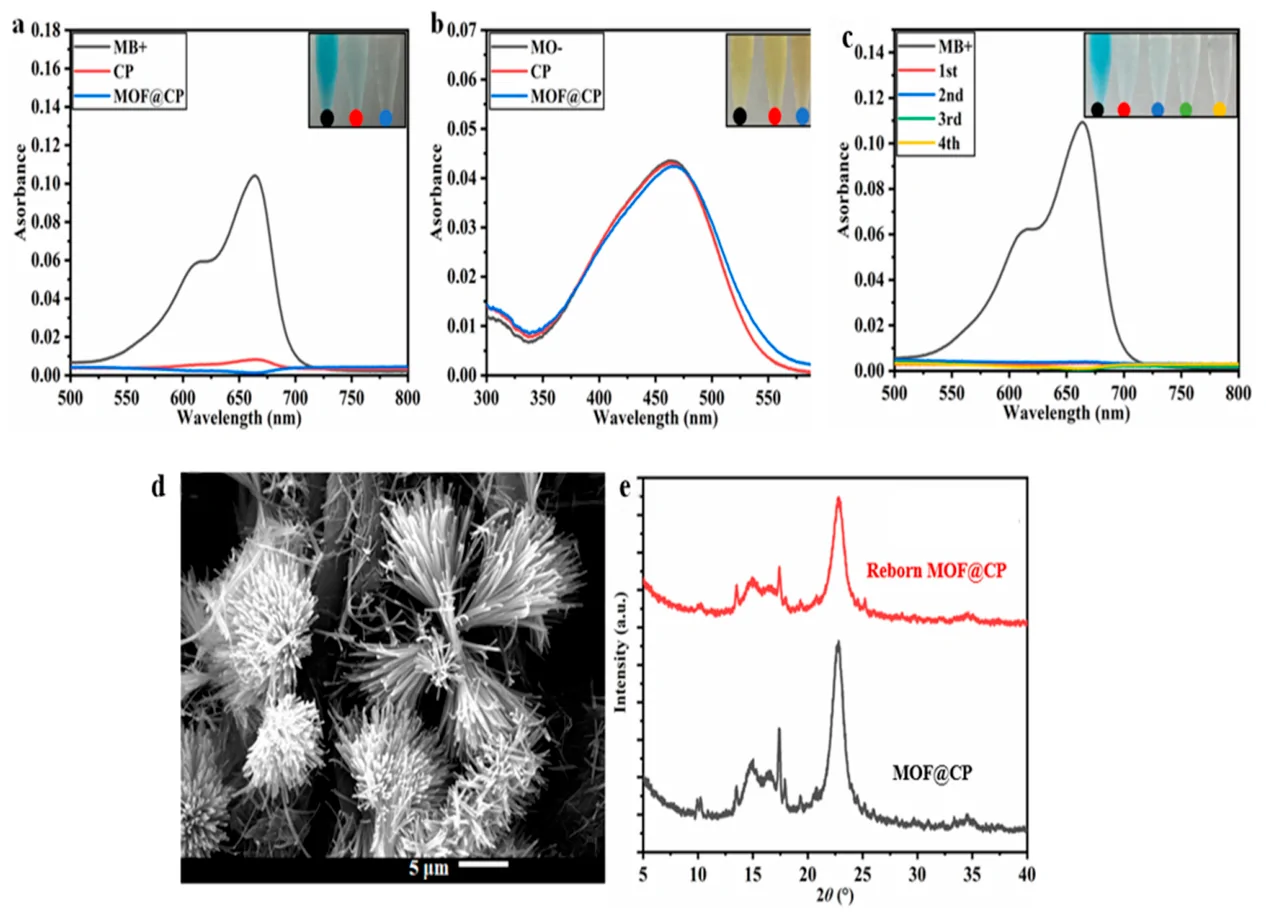
Absorption spectra and color changes in the filtrates obtained after filtering aqueous solutions of MB^+^ (**a**) and MO^−^ (**b**) with CP or Eu_0.1_Tb_0.4_-MOF@CP. MB^+^: Methylene blue; MO^−^: Methyl orange; Dye concentration: 5 ppm; (**c**) Absorption spectra and color changes in the filtrates obtained after four cycles of filtration for MB^+^ adsorption and washing−regeneration of Eu_0.1_Tb_0.4_-MOF@CP; (**d**) SEM image and (**e**) PXRD pattern of the Eu_0.1_Tb_0.4_-MOF@CP regenerated after the fourth washing.

As shown in Figure 6a, after filtration of the MB^+^ solution, the characteristic absorption peak of MB^+^ almost disappeared, and the blue color of the filtrate was significantly weakened. This result indicates that MB^+^ was effectively adsorbed by both Eu_0.1_Tb_0.4_-MOF@CP and CP. The adsorption of MB^+^ by CP can be attributed to the negatively charged cellulose surface, which can interact electrostatically with cationic MB^+^ [50]. In contrast, as shown in Figure 6b, the absorption signal and color of the MO^−^ filtrate changed only slightly after filtration, suggesting that negatively charged MO^−^ was barely adsorbed by either Eu_0.1_Tb_0.4_-MOF@CP or CP. These results demonstrate that the adsorption behavior is mainly governed by electrostatic interactions, and Eu_0.1_Tb_0.4_-MOF@CP exhibits preferential adsorption toward cationic dyes.

To clarify the role of the Eu_0.1_Tb_0.4_-BTC MOF coating in dye adsorption, comparative experiments were performed under varied conditions. The concentration of the cationic dye methylene blue (MB^+^) was first increased from 5 ppm to 50 ppm, and MB^+^ aqueous solution was then replaced with MB^+^ solutions in N,N-dimethylformamide (DMF), acetonitrile, and methanol. As shown in Figure S12, Eu_0.1_Tb_0.4_-MOF@CP showed much stronger MB^+^ adsorption than cellulose filter paper (CP), both for the high-concentration aqueous solution and for the organic solvent systems. This difference was especially pronounced in organic solvents, where CP showed only weak MB^+^ adsorption (Figure S12b–d).

The enhanced adsorption can be mainly attributed to two factors. First, the Eu_0.1_Tb_0.4_-BTC MOF coating provides more negative charges than bare CP, strengthening the electrostatic interaction with MB^+^. Second, in organic solvents, the ionization of acidic groups on CP, such as -OH and a small amount of -COOH, is suppressed, reducing the surface negative charge and weakening its affinity for MB^+^.

**Figure 7:**
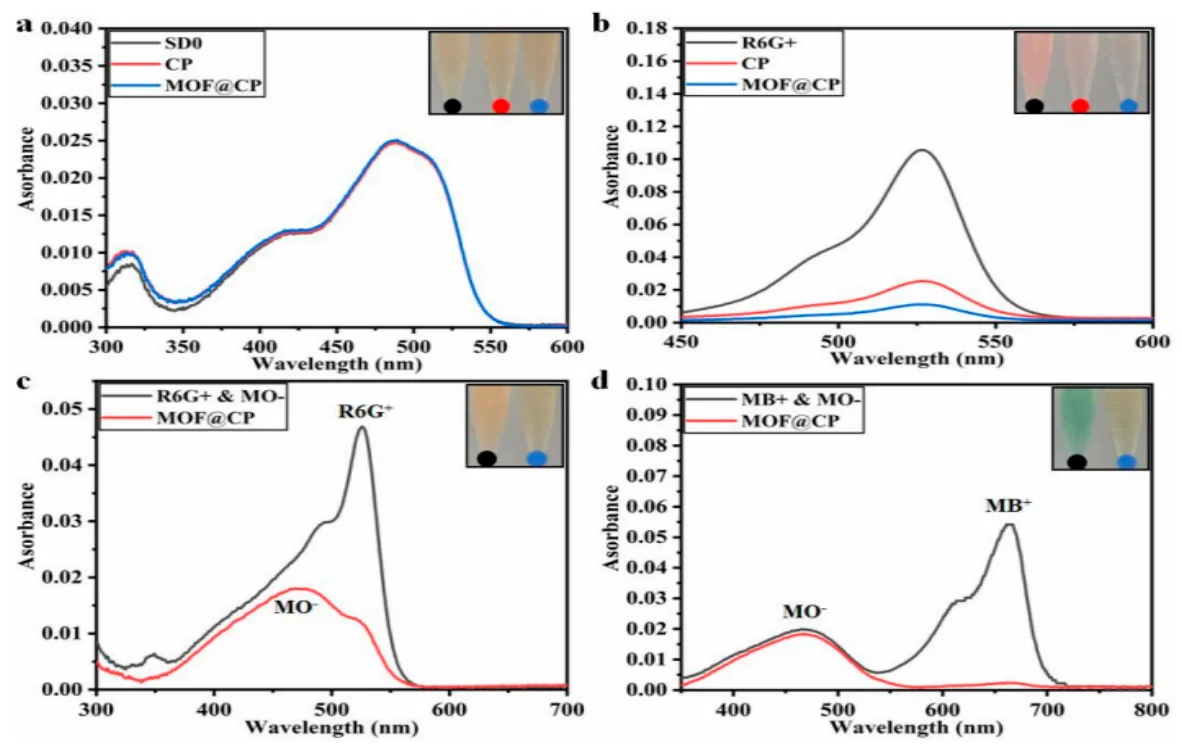
Absorption spectra and color changes in the filtrates obtained after filtering solutions of SD_0_ (**a**), R6G^+^ (**b**), MO^−^ and R6G^+^ (**c**), MO^−^ and MB^+^ (**d**) with CP or Eu_0.1_Tb_0.4_-MOF@CP. Dye concentration: 5 ppm.

Selective adsorption is an important criterion for evaluating adsorption materials [50,51]. To further investigate the charge-selective adsorption behavior of Eu_0.1_Tb_0.4_-MOF@CP, methylene blue (MB^+^), rhodamine 6G (R6G^+^), methyl orange (MO^−^), and Sudan 0 (SD_0_) were selected as representative competitive dyes. As expected, the neutral dye SD_0_ was barely adsorbed by Eu_0.1_Tb_0.4_-MOF@CP, as shown in Figure 7a, whereas the cationic dye R6G^+^ was efficiently removed by the material, as shown in Figure 7b.

The selectivity of Eu_0.1_Tb_0.4_-MOF@CP was further verified using binary dye mixtures. As shown in Figure 7c,d, after filtration through Eu_0.1_Tb_0.4_-MOF@CP, the characteristic absorption peaks of MB^+^ and R6G^+^ almost disappeared, while the absorption intensity of MO^−^ remained nearly unchanged. Eu_0.1_Tb_0.4_-MOF@CP selectively captures cationic dyes from mixed-dye systems, with negligible interference from neutral or anionic dyes.

This selective removal process can also be directly observed by the naked eye through the color change in the filtrates. For example, the original orange-red mixed solution of MO^−^ and R6G^+^ turned orange after filtration because R6G^+^ was effectively captured by the fluorescent paper, as shown in the inset of Figure 7c. Similarly, after filtration of the MO^−^/MB^+^ mixed solution, the solution color changed from green to orange due to the selective removal of MB^+^, as shown in the inset of Figure 7d.

Overall, these results demonstrate that Eu_0.1_Tb_0.4_-MOF@CP exhibits excellent charge-selective adsorption ability and can serve as an effective paper-based medium for the selective removal of cationic dyes from aqueous solutions.

Recyclability is another important parameter for evaluating adsorption materials [50]. To assess the reusability of Eu_0.1_Tb_0.4_-MOF@CP, the dye-loaded paper was regenerated by washing with saturated sodium chloride solution in N,N-dimethylformamide (DMF) [52], followed by repeated adsorption experiments. The adsorption performance of Eu_0.1_Tb_0.4_-MOF@CP could be effectively recovered after the washing treatment. As shown in Figure 6c, Eu_0.1_Tb_0.4_-MOF@CP still maintained good adsorption ability after four adsorption-regeneration cycles.

The structural stability of the regenerated material was further investigated by SEM and PXRD. The SEM image in Figure 6d shows that the Eu_0.1_Tb_0.4_-BTC MOF particles remained attached to the cellulose surface after four regeneration cycles, showing that MOF particles remained observable on the cellulose surface after four regeneration cycles. Together with the retained characteristic PXRD reflections and adsorption response, these results support morphological, crystalline, and functional retention under the tested regeneration conditions. In addition, the PXRD pattern in Figure 6e confirms that the regenerated Eu_0.1_Tb_0.4_-MOF@CP preserved the characteristic crystalline structure of the Eu_0.1_Tb_0.4_-MOF coating. These results suggest that repeated adsorption and regeneration did not cause obvious structural damage to the material.

Therefore, Eu_0.1_Tb_0.4_-MOF@CP exhibits not only charge-selective adsorption performance but also good recyclability and structural stability, demonstrating its potential as a reusable paper-based adsorbent for practical dye-removal applications.

### 3.9. Universality of the Preparation Method

Previous results confirmed that Ln-BTC MOF fluorescent coatings could be constructed on cellulose paper by liquid-phase epitaxial layer-by-layer growth and applied to visual fluorescence sensing and selective dye adsorption. However, this result alone does not prove that the method is applicable to other Ln-MOF systems. To evaluate its universality, Ln-BDC MOF@CP and Ln-BPDC MOF@CP were prepared using the same strategy, where Ln represents Eu and Tb, BDC is terephthalic acid, and BPDC is 4,4′-biphenyldicarboxylic acid.

Figures S13 and S14 show the excitation and emission spectra of Eu-BDC MOF@CP, Tb-BDC MOF@CP, Eu-BPDC MOF@CP, and Tb-BPDC MOF@CP, respectively. Upon excitation, these fluorescent papers show the characteristic emissions of Eu^3+^ or Tb^3+^, giving red Eu^3+^ fluorescence or green Tb^3+^ fluorescence, as shown in the upper insets of Figures S13a,b and S14a,b. All four Ln-MOF@CP samples also retain the flexibility and cuttability of cellulose filter paper, as shown in the lower insets of Figures S13a,b and S14a,b.

FT-IR spectra (Figures S13c and S14c) and PXRD patterns (Figures S13d and S14d) confirm that Ln-BDC MOF and Ln-BPDC MOF (Ln = Eu^3+^ or Tb^3+^) grow layer by layer on CP while maintaining their chemical and crystal structures. SEM images (Figure S15) further show that the four fluorescent papers possess uniformly distributed nanoscale flower-like Ln-MOF crystals on the cellulose surface.

The successful preparation of different Ln-MOF fluorescent papers confirms the general applicability of the liquid-phase epitaxial strategy. Different Ln-MOFs can be used as luminescent coatings to prepare fluorescent papers with various functions and orientations. This advantage can be attributed to the large number of active oxygen-containing functional groups (such as carbonyl, carboxyl, and hydroxyl groups) on the surface of the cellulose paper [53], which can effectively control the growth orientation of the Ln-MOF fluorescent layer [21,54]. By using the liquid-phase epitaxial layer-by-layer growth method, not only can the thickness of the fluorescent coating be precisely controlled, but also the growth orientation of Ln-MOFs can be adjusted. Moreover, the prepared fluorescent papers have uniform and stable fluorescence properties, which opens up new prospects for the development of high-performance light-emitting devices and fluorescent sensors.

## 4. Conclusions

In summary, a stable Ln-MOF/cellulose composite material was successfully fabricated through a binder-free liquid-phase epitaxial growth strategy on unmodified cellulose fibers. This approach enabled the direct construction of uniformly distributed Ln-MOF coatings while preserving the flexibility and processability of the cellulose substrate. The optimized composite exhibited homogeneous luminescence with low relative standard deviations (<2%) and excellent fluorescence stability over a broad pH range (3–11), demonstrating the advantages of LPE growth in constructing reliable cellulose-based functional materials. Moreover, the universality of this strategy was confirmed by extending the preparation from Ln-BTC to Ln-BDC and Ln-BPDC systems, indicating its potential applicability for constructing diverse Ln-MOF/cellulose composites.

The functional properties of the obtained composite material were further demonstrated through optical sensing and selective separation applications. By regulating the Eu^3+^/Tb^3+^ emission balance, the composite enabled smartphone-assisted ratiometric visualization of dipicolinic acid with a linear response range of 0–2000 μM and a detection limit of 10 μM. In addition, the anionic Ln-MOF coating enhanced the charge-selective adsorption capability of cellulose, allowing preferential removal of cationic dyes while maintaining structural integrity after four regeneration cycles. These results highlight that LPE provides an effective route for integrating functional MOF coatings with sustainable cellulose substrates and offers a general strategy for developing stable cellulose-based composite materials with potential applications in chemical sensing and environmental remediation.

## Data Availability

All data will be made available upon request to the corresponding author.

## Supplementary Materials

The following supporting information can be downloaded at: https://www.mdpi.com/article/10.3390/polym18172178/s1, Figure S1: Fluorescence images (λ_ex_ = 254 nm) (a) and PXRD patterns (b) of Tb-MOF@CP prepared by different methods. Methods 1–5: In-situ growth method; Methods 6–9: Liquid - phase epitaxial method; Figure S2: SEM images of Tb-MOF@CP prepared by different methods. (a) Method 1; (b1), (b2) Method 6; (c1), (c2) Method 7; (d1), (d2) Method 8; (e1), (e2) Method 9; Figure S3: Fluorescence spectra (a) and fluorescence intensities (b) of Tb - MOF@CP prepared with different numbers of cycles. The inset in Figure a shows the corresponding images of Tb-MOF@CP under a 254-nm ultraviolet lamp; Figure S4: SEM images of Tb-MOF@CP prepared with different numbers of cycles. (a) 2 cycles; (b) 4 cycles; (c1), (c2) 8 cycles; (d) 12 cycles; (e) 16 cycles; Figure S5: Fluorescence spectra (λ_ex_ = 270 nm) of three different points on Tb-MOF@CP (a) and three Tb-MOF@CP samples prepared in different batches (b). The insets show the corresponding fluorescence intensities of Tb^3+^ at 546 nm for Tb-MOF@CP; Figure S6: Fluorescence intensities of (a) Tb-BTC MOF powder (500 μg/mL), (b) Tb-MOF@CP prepared by the soaking method, and (c) Tb-MOF@CP prepared by the liquid-phase epitaxial method in water within 30 min (λ_ex_ = 270 nm, λ_em_ = 546 nm), (d) Nitrogen adsorption-desorption isotherms and the corresponding BJH pore-size distribution curves (inset) of Eu_0.1_Tb_0.4_-MOF@CP samples; Figure S7: (a) Schematic diagram of the ligand-to-metal energy transfer from BTC to Ln^3+^ and the metal-to-metal energy transfer from Tb^3+^ to Eu^3+^; (b) Results of the calculation of the BTC ligand using the B_3_LYP/6-31G* method in density functional theory; Figure S8: (a) Solid-state emission spectra of Eu_x_Tb_1−x_-MOF@CP (x = 1, 0.3, 0.2, 0.15, 0.1, 0.05, 0.01 and 0) (λ_ex_ = 278 nm), and the inset is the corresponding image under a 254 nm ultraviolet lamp; (b) CIE chromaticity diagram of Eu_x_Tb_1−x_-MOF@CP (x = 1, 0.3, 0.2, 0.15, 0.1, 0.05, 0.01 and 0); Figure S9: (a) Fluorescence image of a circular paper piece (Eu_0.1_Tb_0.4_-MOF@CP, with a diameter of 6 mm) under a 254 nm ultraviolet lamp; (b) pH stability study of Eu_0.1_Tb_0.4_-MOF@CP. The inset shows the fluorescence image under a 254 nm ultraviolet lamp; Figure S10: Fluorescence decay curves (λ_ex_ = 270 nm) monitoring the emission of the ^5^D_0_ → ^7^F_2_ transition at 618 nm (a) and the ^5^D_4_ → ^7^F_5_ transition at 546 nm (b) of Eu_0.1_Tb_0.4_-MOF@CP; Figure S11: (a) FT-IR spectra and (b) PXRD patterns of Eu_0.1_Tb_0.4_-MOF@CP after treatment with aqueous solutions (10 mM) of Fe^3+^, CO_3_^2−^, or PO_4_^3−^; Figure S12: Absorption spectra and color changes of the filtrates obtained after filtering solutions of MB^+^ in water (a), DMF (b), acetonitrile (c), and methanol (d) with CP or Eu_0.1_Tb_0.4_-MOF@CP. Dye concentration in water: 50 ppm; Dye concentration in DMF, acetonitrile, and methanol: 5 ppm; Figure S13: (a) Excitation and emission spectra of Eu-BDC MOF@CP and (b) those of Tb-BDC MOF@CP. The insets are the fluorescence images (λ_ex_ = 254 nm) and the macroscopic folded views; (c) FT-IR spectra and (d) PXRD spectra of Eu-BDC MOF@CP and Tb-BDC MOF@CP; Figure S14: (a) Excitation and emission spectra of Eu-BPDC MOF@CP and (b) those of Tb-BPDC MOF@CP. The insets are the fluorescence images (λ_ex_ = 254 nm) and the macroscopic folded views; (c) FT-IR spectra and (d) PXRD spectra of Eu-BPDC MOF@CP and Tb-BPDC MOF@CP; Figure S15: Scanning electron microscopy images of (a) Eu-BDC MOF@CP, (b) Tb-BDC MOF@CP, (c) Tb-BPDC MOF@CP, and (d) Eu-BPDC MOF@CP; Figure S16: (a) Relative G/R value of Eu_0.1_Tb_0.4_-MOF@CP upon addition of DPA, (b) Corresponding fluorescence images under 254 nm UV lamp.All substances were tested at a final concentration of 2000 uM in aqueous solution; Table S1: Methods and Conditions for Preparing Tb-MOF@CP Tb^3+^: Tb(NO_3_)_3_•6H_2_O; r.t.: Room temperature; time: Reaction time; times: Number of cycles; washed: Washed during the cycling process; Methods 1–5: In-situ growth method; Methods 6–9: Liquid-phase epitaxial method.
