# Supplementary material for: LPE-Grown Lanthanide-MOF/Cellulose Paper for Visual Sensing and Selective Dye Removal
Source: Polymers (Basel). 2026 Sep 7;18(17):2178. doi: 10.3390/polym18172178 (PMC13567998; doi:10.3390/polym18172178)
Supplement: Supplementary file 1 [file polymers-18-02178-s001.zip › polymers-4509601-supplementary.pdf]

Supplementary Material

S1. Materials

1,3,5-Benzenetricarboxylic acid (H_3BTC) was purchased from Shanghai Macklin Biochemical Technology Co, Ltd(No. 169, Chugong Road, Fengxian District, Shanghai). 4,4'-Biphenyldicarboxylic acid (H_2BPDC), europium(III) nitrate hexahydrate ($Eu(NO_3)_3·6H_2O$), terbium(III) nitrate hexahydrate ($Tb(NO_3)_3·6H_2O$), and pyridine-2,6-dicarboxylic acid (DPA) were purchased from Sain Chemical Technology (Shanghai) Co, Ltd(Room 387, Building 5, No. 2278, Zhaozhong Road, Chonggu Town, Qingpu District, Shanghai). Dyes, metal ion nitrates, anionic sodium salts, N,N-dimethylformamide (DMF), ethanol, etc. were all purchased from Sinopharm Chemical Reagent Co, Ltd(Building 1, No. 801, Hutai Road, Jing 'an District, Shanghai). Terephthalic acid (H_2BDC) was purchased from Aladdin Reagent (Shanghai) Co., Ltd(No. 809, Chuhua Branch Road, Fengxian District, Shanghai). All of the above reagents were of analytical grade and did not require further purification. Cellulose filter paper (Shuangquan qualitative filter paper) was purchased from GE Healthcare Bio-Sciences (Shanghai) Co, Ltd(2nd Floor, No. 1, Newton Road, Zhang Jiang Hi-Tech Park, Pudong, Shanghai, China). The deionized water used in the experiments was obtained by treatment with a Millipore instrument (Molsheim, France).

S2. Instrument

The powder X-ray diffraction pattern (PXRD) was obtained on a Bruker D8 diffractometer (Bruker AXS, Germany), and a copper target (40 mA, 40 kV) was used during the measurement. The excitation and emission spectra were measured by a Hitachi F-4600 fluorescence spectrophotometer (Hitachi High-Technologies Corporation, Japan), with a 450-watt xenon lamp as the excitation source. The Fourier transform infrared (FT-IR) spectrum of the powder was obtained on a Thermo Nicolet NEXUS 470 infrared spectrometer (Thermo Fisher Scientific, USA), and the measurement was carried out by the method of grinding the material with potassium bromide and pressing it into a tablet. The infrared spectrum of the fluorescent paper

was obtained on a NICOLET 5700 Fourier transform infrared spectrometer (Thermo Fisher Scientific, USA). The ultraviolet-visible absorption spectrum was measured using a Shimadiu UV-2600 ultraviolet spectrophotometer (Shimadzu Corporation, Japan). The scanning electron microscope (SEM) images were obtained on a JSM-6510 scanning electron microscope (JEOL Ltd., Japan). The EDS (Energy Dispersive Spectrometry) elemental distribution map was measured by a JSM-7500F field emission scanning electron microscope equipped with an X-maxN80 energy dispersive spectrometer (JEOL Ltd., Japan). The XPS spectrum was obtained by a Thermo Scientific K-Alpha X-ray photoelectron spectrometer (Thermo Fisher Scientific, USA). The fluorescent images were taken with a smartphone under the illumination of a ZF-7A portable ultraviolet lamp.

S3.Optimization of Preparation Conditions

Method	Ln^{3+}	Ligand	Solvent	Additive	Temperature	Time/Times
1	Tb^{3+}	H_3BTC	DMF/EtOH	-	80 °C	24 h
2	Tb^{3+}	H_3BTC	DMF /H_2O	HNO_3	80 °C	24 h
3	Tb^{3+}	H_3BTC	DMF /H_2O	NaOH	r.t.	3 h
4	Tb^{3+}	H_3BTC	DMF /H_2O	CH_3COONa	r.t.	3 h
5	Tb^{3+}	H_3BTC	EtOH/H_2O	-	r.t.	3 h
6	Tb^{3+}	H_3BTC	EtOH/H_2O	CH_3COONa	r.t.	12 times/unwashed
7	Tb^{3+}	H_3BTC	EtOH/H_2O	CH_3COONa	r.t.	12 times/washed
8	Tb^{3+}	H_3BTC	EtOH/H_2O	-	r.t.	12 times/unwashed
9	Tb^{3+}	H_3BTC	EtOH/H_2O	-	r.t.	12 times/washed

Table S1 Methods and Conditions for Preparing Tb-MOF@CP Tb^{3+}: $Tb(NO_3)_3•6H_2O$; r.t.: Room temperature; time: Reaction time; times: Number of cycles; washed: Washed during the cycling process; Methods 1-5: In-situ growth method;Methods 6-9: Liquid-phase epitaxial method.

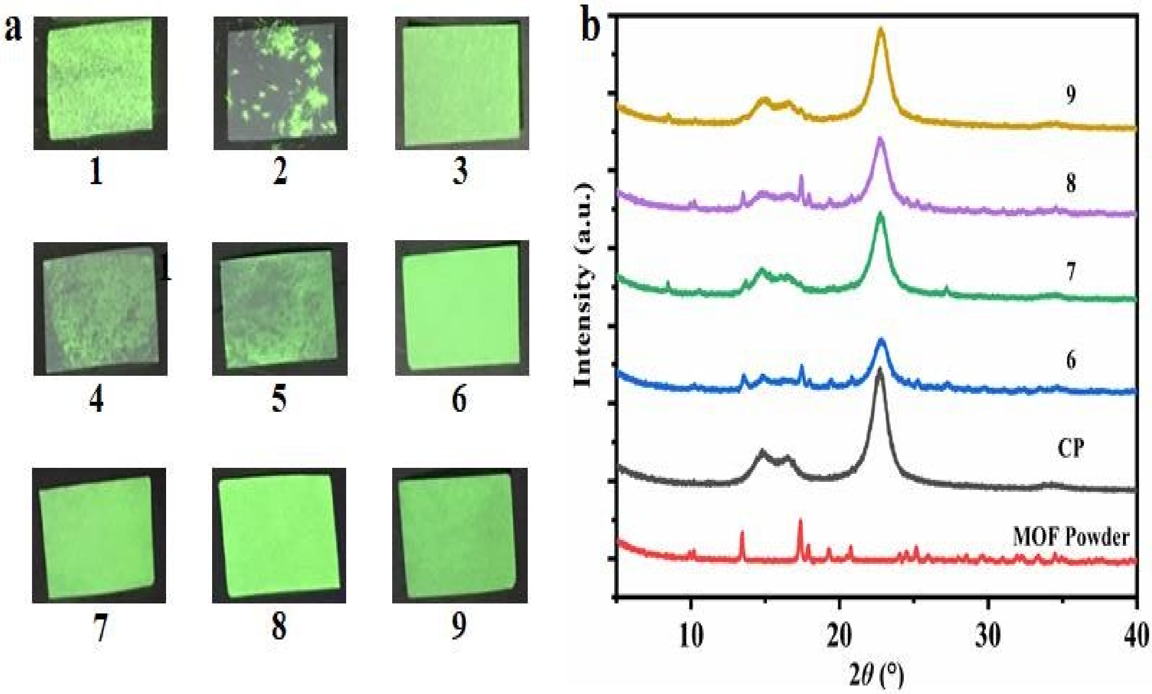

Figure S1 Fluorescence images (λ_{ex} = 254 nm) (a) and PXRD patterns (b) of Tb-MOF@CP prepared by different methods. Methods 1-5: In-situ growth method; Methods 6-9: Liquid - phase epitaxial method.

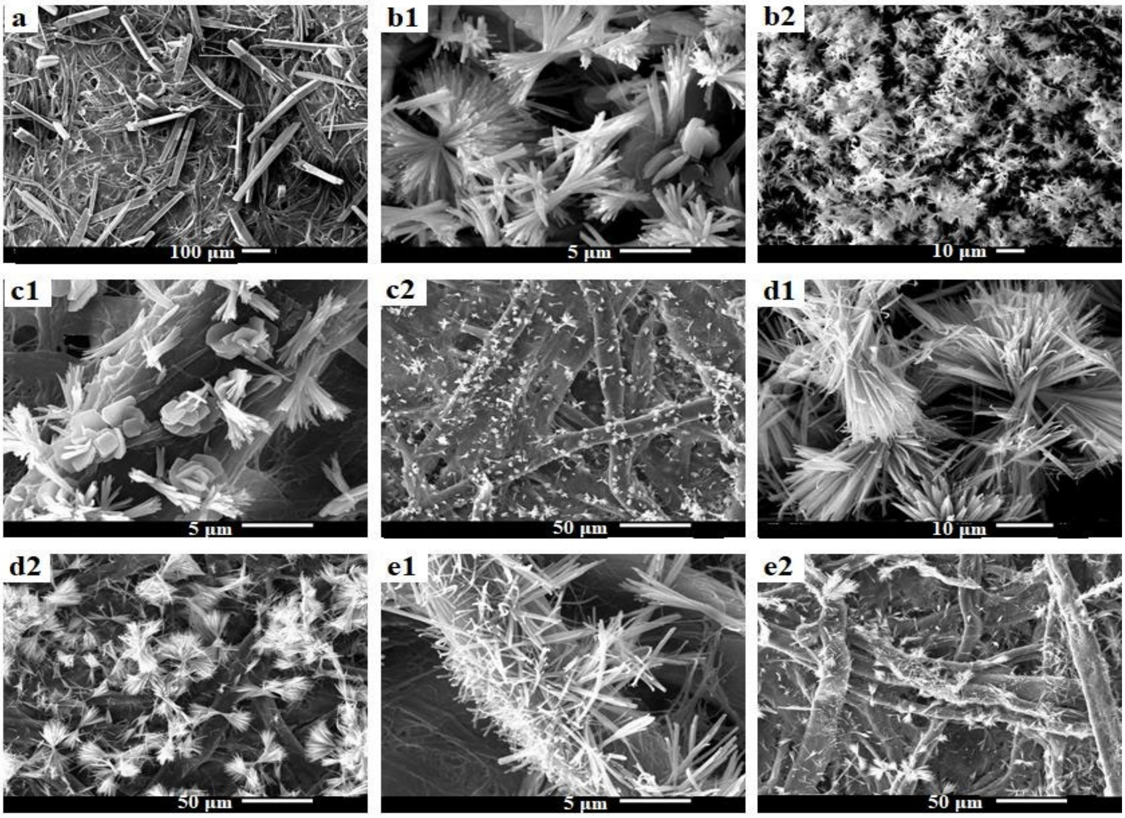

Figure S2 SEM images of Tb-MOF@CP prepared by different methods. (a) Method 1; (b1), (b2) Method 6; (c1), (c2) Method 7; (d1), (d2) Method 8; (e1), (e2) Method 9.

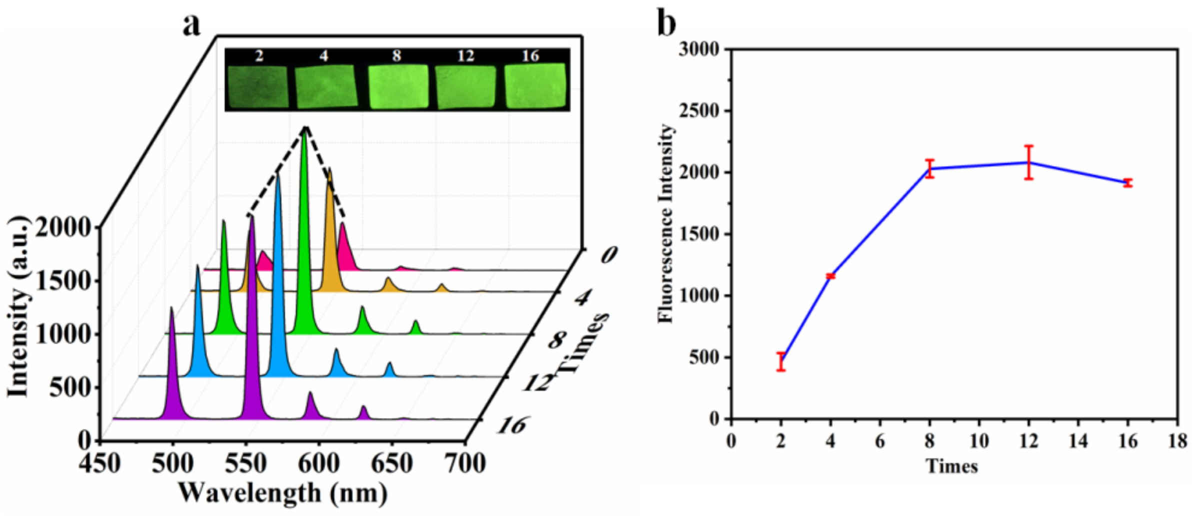

Figure S3 Fluorescence spectra (a) and fluorescence intensities (b) of Tb - MOF@CP prepared with different numbers of cycles. The inset in Figure a shows the corresponding images of Tb-MOF@CP under a 254-nm ultraviolet lamp.

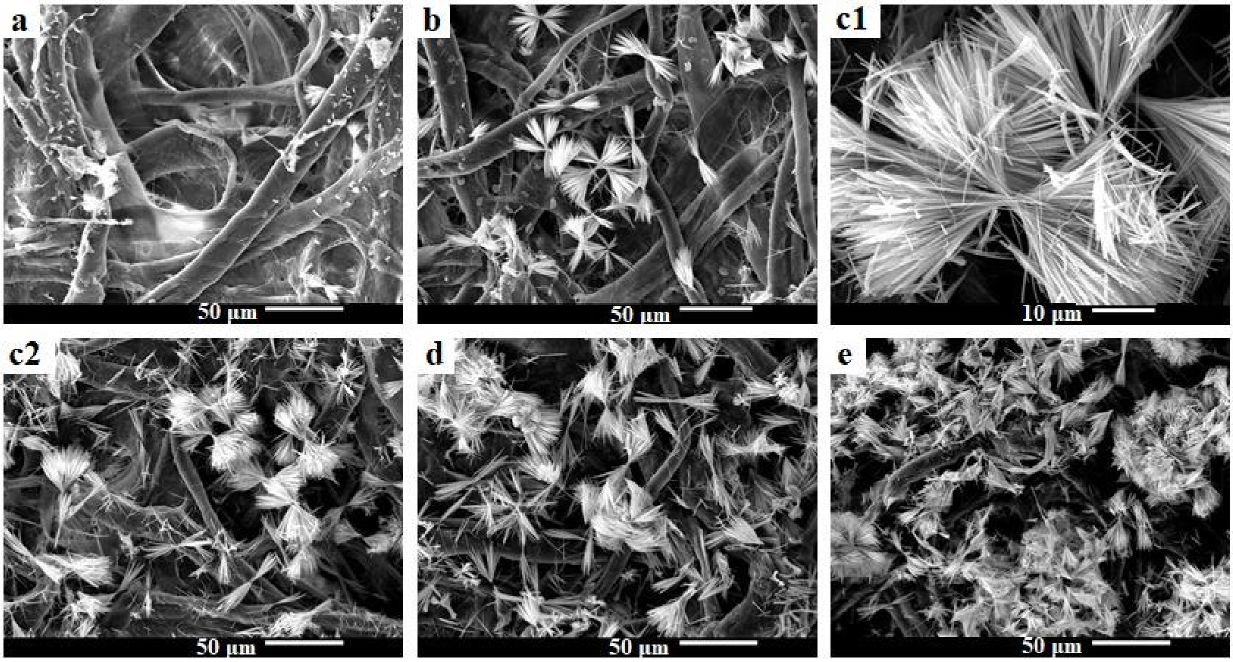

Figure S4 SEM images of Tb-MOF@CP prepared with different numbers of cycles. (a) 2 cycles; (b) 4 cycles; (c1), (c2) 8 cycles; (d) 12 cycles; (e) 16 cycles.

S4. Homogeneity and Stability

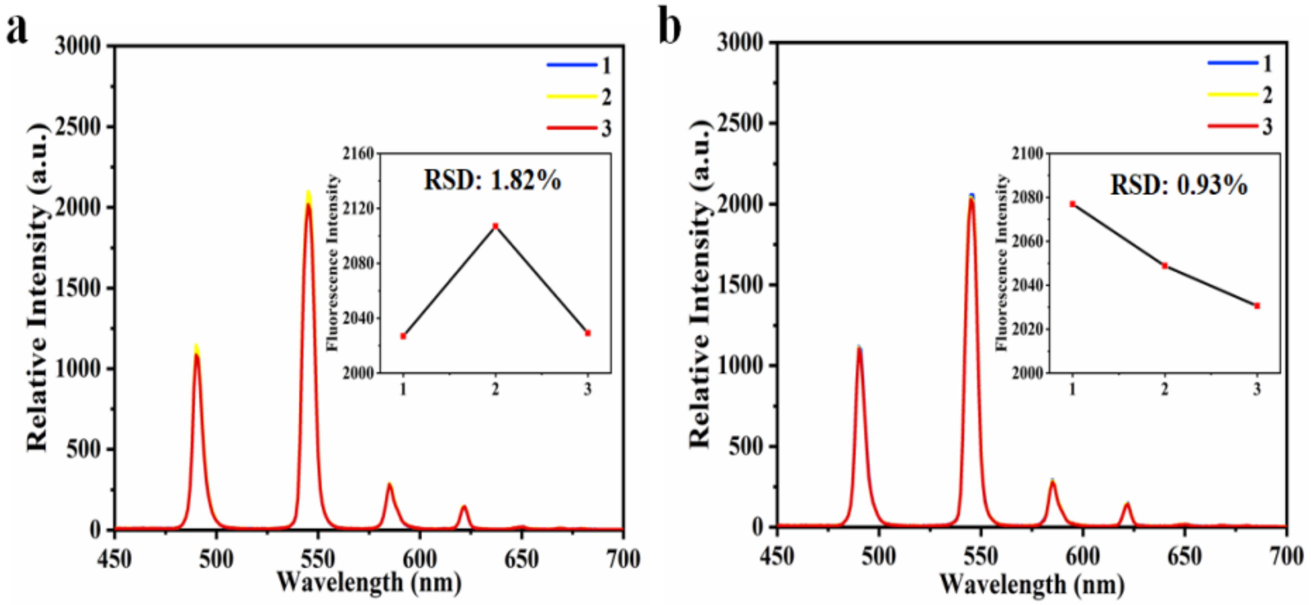

Figure S5 Fluorescence spectra (λ_{ex} = 270 nm) of three different points on Tb-MOF@CP (a) and three Tb-MOF@CP samples prepared in different batches (b). The insets show the corresponding fluorescence intensities of Tb^{3+} at 546 nm for Tb-MOF@CP.

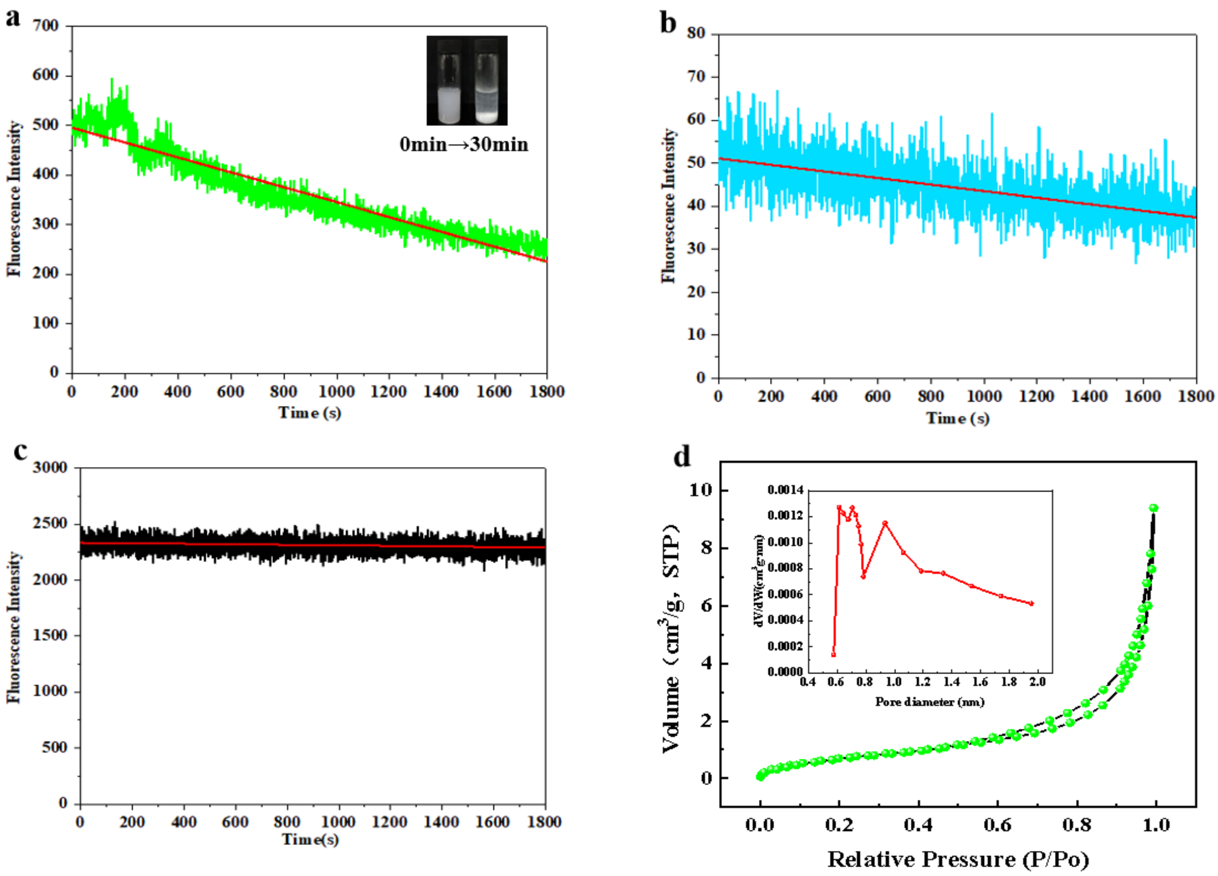

Figure S6 Fluorescence intensities of (a) Tb-BTC MOF powder (500 μg/mL), (b) Tb-MOF@CP prepared by the soaking method, and (c) Tb-MOF@CP prepared by the liquid-phase epitaxial method in water within 30 min (λ_{ex} = 270 nm, λ_{em} = 546 nm), (d) Nitrogen adsorption-desorption isotherms and the corresponding BJH pore-size distribution curves (inset) of $Eu_{0.1}Tb_{0.4}$-MOF@CP samples.

S5. Luminescent Properties

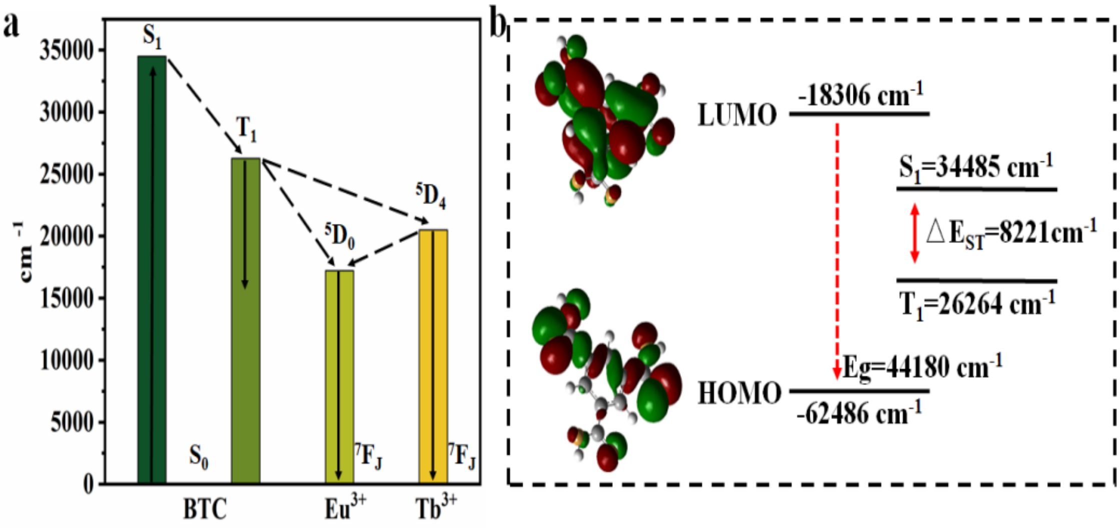

Figure S7 (a) Schematic diagram of the ligand-to-metal energy transfer from BTC to Ln^{3+} and the metal-to-metal energy transfer from Tb^{3+} to Eu^{3+}; (b) Results of the calculation of the BTC ligand using the $B_3LYP/6\text{-}31G^*$ method in density functional theory.

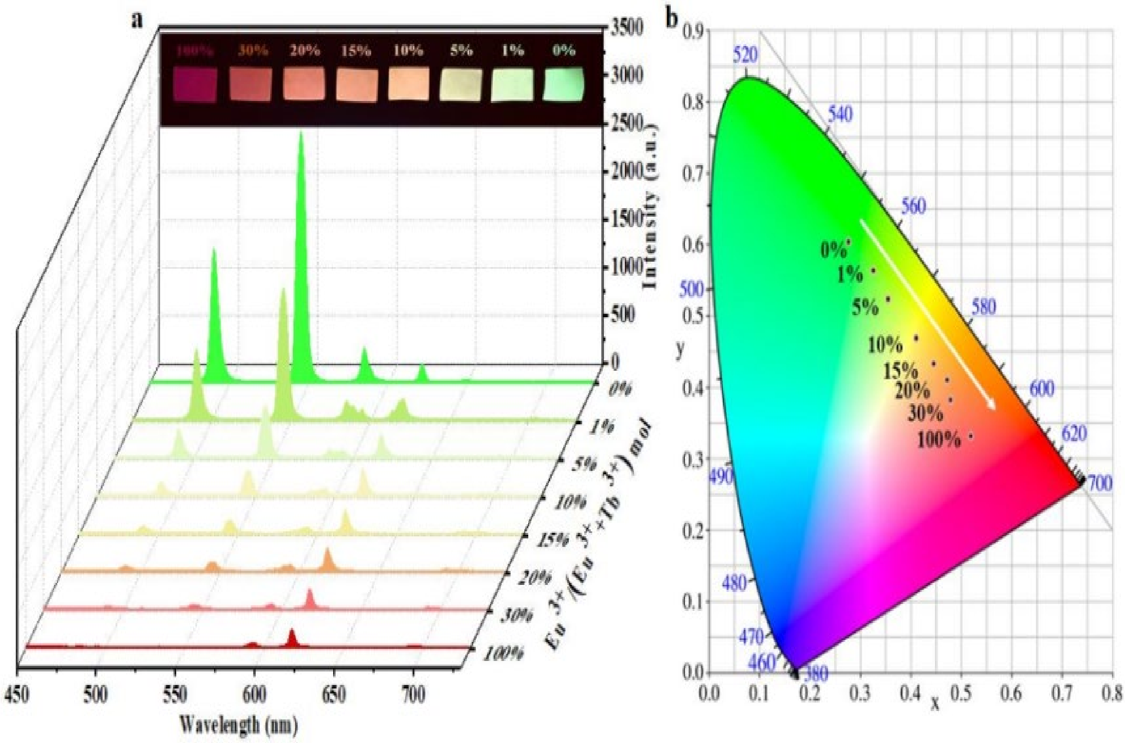

Figure S8 (a) Solid-state emission spectra of Eu_xTb_{1-x}-MOF@CP (x = 1, 0.3, 0.2, 0.15, 0.1, 0.05, 0.01 and 0) (λ_{ex} = 278 nm), and the inset is the corresponding image under a 254 nm ultraviolet lamp; (b) CIE

chromaticity diagram of Eu_xTb_{1-x}-MOF@CP (x = 1, 0.3, 0.2, 0.15, 0.1, 0.05, 0.01 and 0).

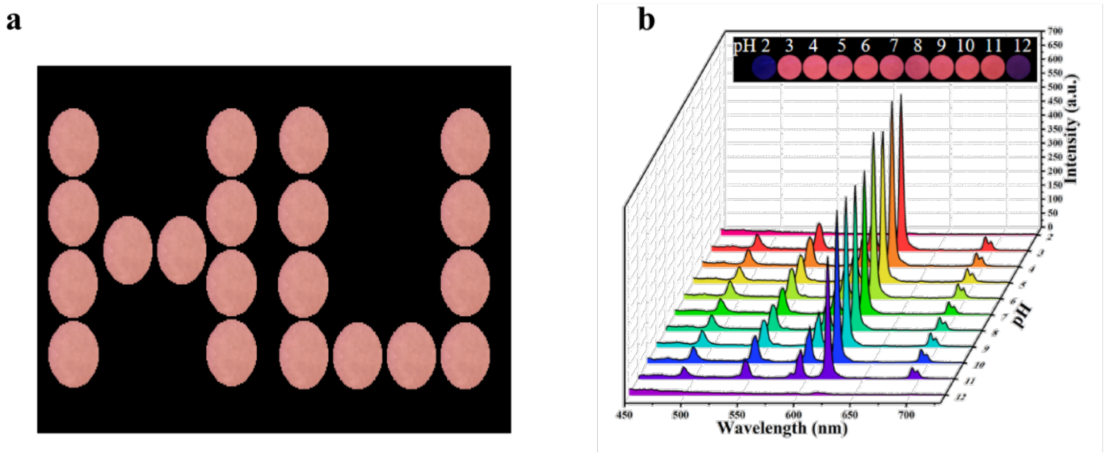

Figure S9 (a) Fluorescence image of a circular paper piece ($Eu_{0.1}Tb_{0.4}$-MOF@CP, with a diameter of 6 mm) under a 254 nm ultraviolet lamp; (b) pH stability study of $Eu_{0.1}Tb_{0.4}$-MOF@CP. The inset shows the fluorescence image under a 254 nm ultraviolet lamp.

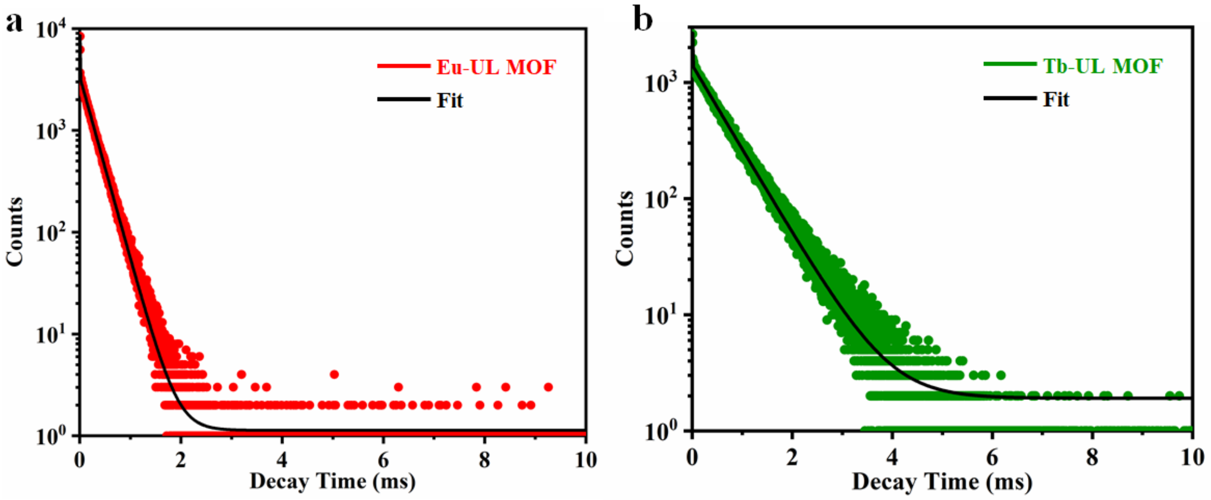

Figure S10 Fluorescence decay curves (λ_{ex} = 270 nm) monitoring the emission of the $^5D_0 \rightarrow {}^7F_2$ transition at 618 nm (a) and the $^5D_4 \rightarrow {}^7F_5$ transition at 546 nm (b) of $Eu_{0.1}Tb_{0.4}$-MOF@CP.

S6. Sensing Mechanism

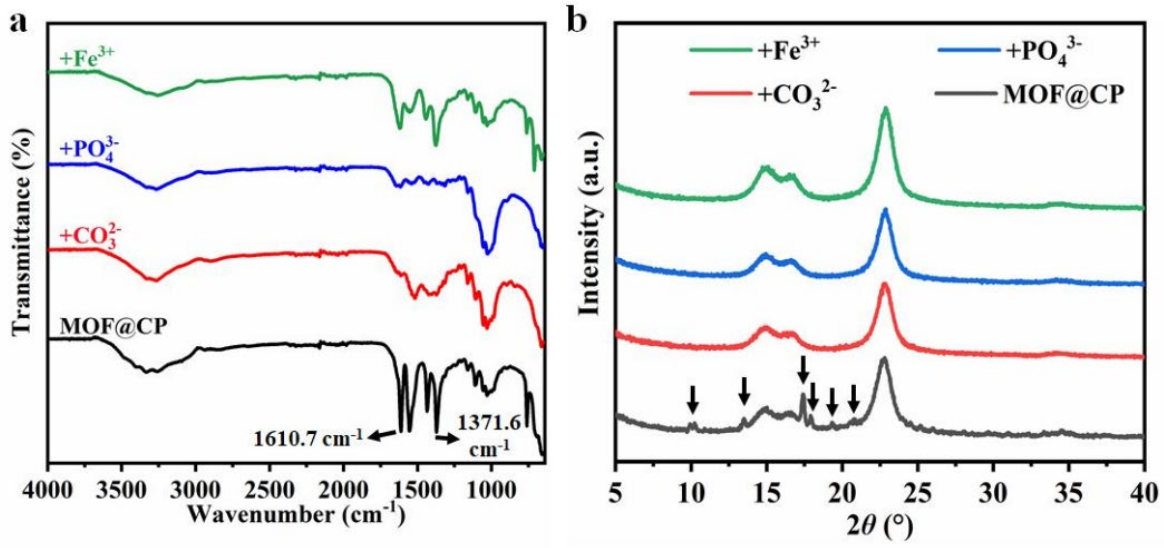

Figure S11 (a) FT-IR spectra and (b) PXRD patterns of $Eu_{0.1}Tb_{0.4}$-MOF@CP after treatment with aqueous solutions (10 mM) of Fe^{3+}, CO_3^{2-}, or PO_4^{3-}.

S7. Selective Removal of Dyes

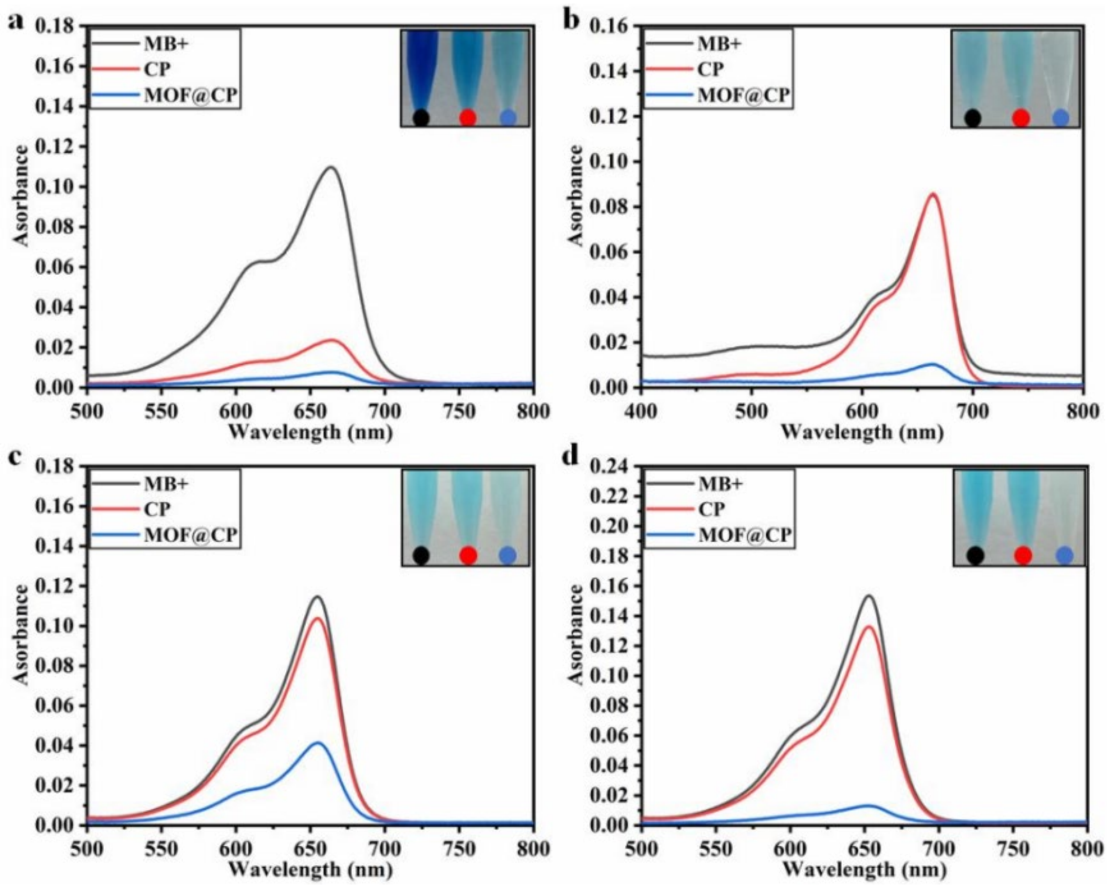

Figure S12 Absorption spectra and color changes of the filtrates obtained after filtering solutions of MB^+ in water (a), DMF (b), acetonitrile (c), and methanol (d) with CP or $Eu_{0.1}Tb_{0.4}$-MOF@CP. Dye concentration in water: 50 ppm; Dye concentration in DMF, acetonitrile, and methanol: 5 ppm.

S8. Universality of the preparation method

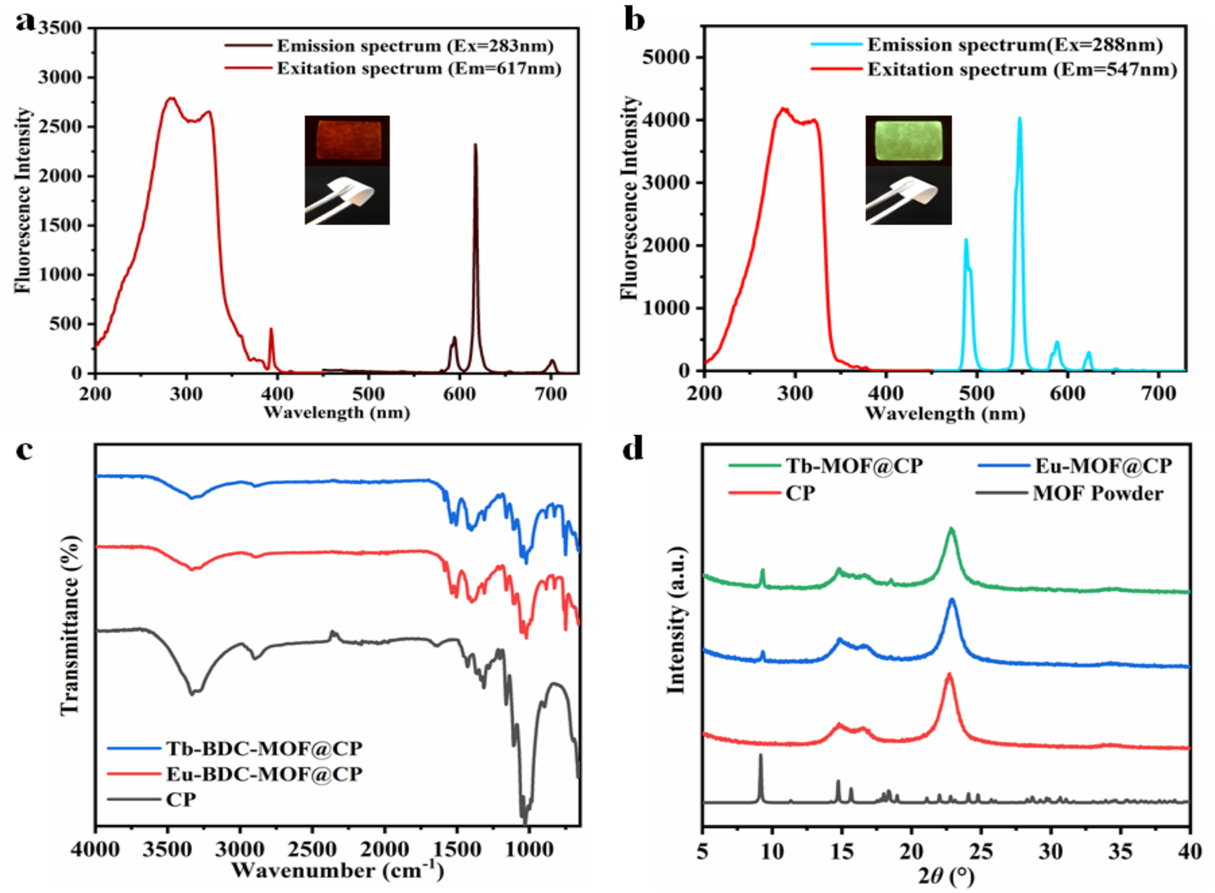

Figure S13 (a) Excitation and emission spectra of Eu-BDC MOF@CP and (b) those of Tb-BDC MOF@CP. The insets are the fluorescence images (λ_{ex} = 254 nm) and the macroscopic folded views; (c) FT-IR spectra and (d) PXRD spectra of Eu-BDC MOF@CP and Tb-BDC MOF@CP.

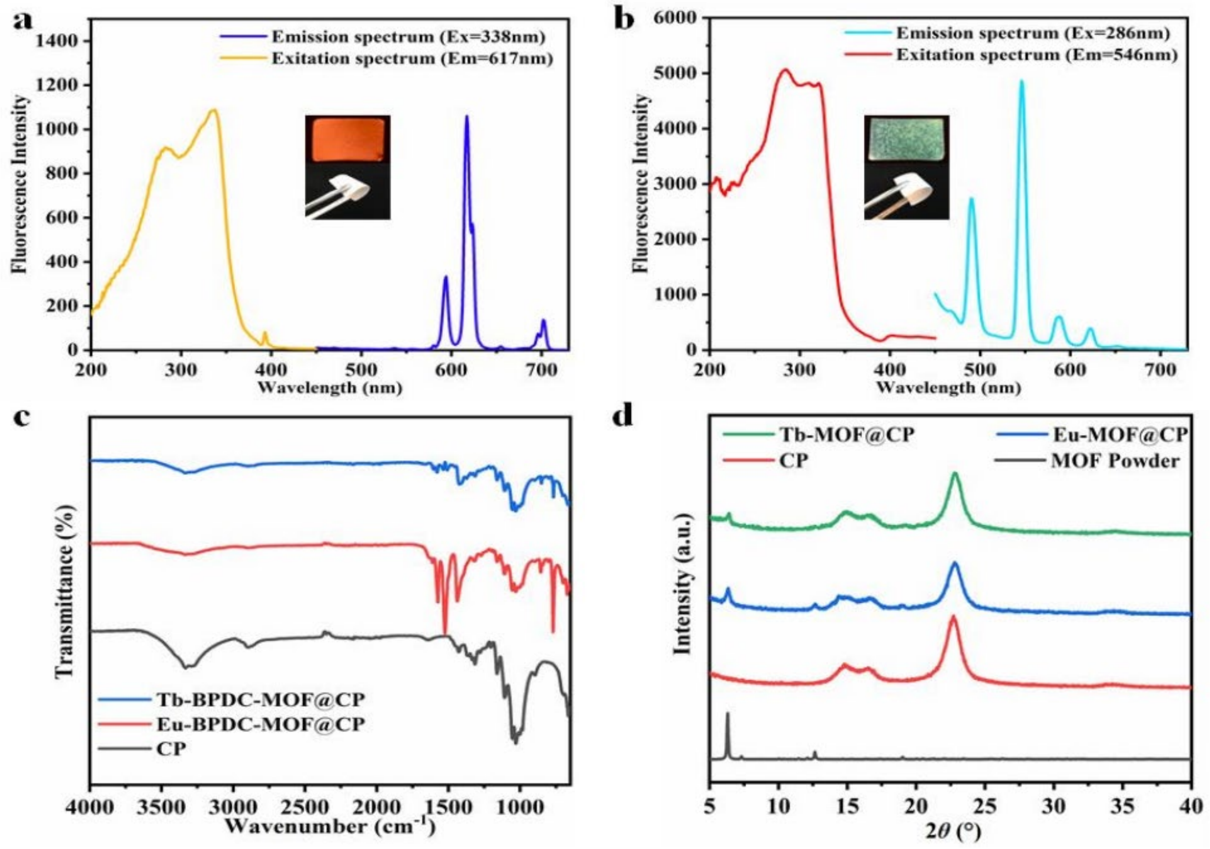

Figure S14 (a) Excitation and emission spectra of Eu-BPDC MOF@CP and (b) those of Tb-BPDC MOF@CP. The insets are the fluorescence images (λ_{ex} = 254 nm) and the macroscopic folded views; (c) FT-IR spectra and (d) PXRD spectra of Eu-BPDC MOF@CP and Tb-BPDC MOF@CP.

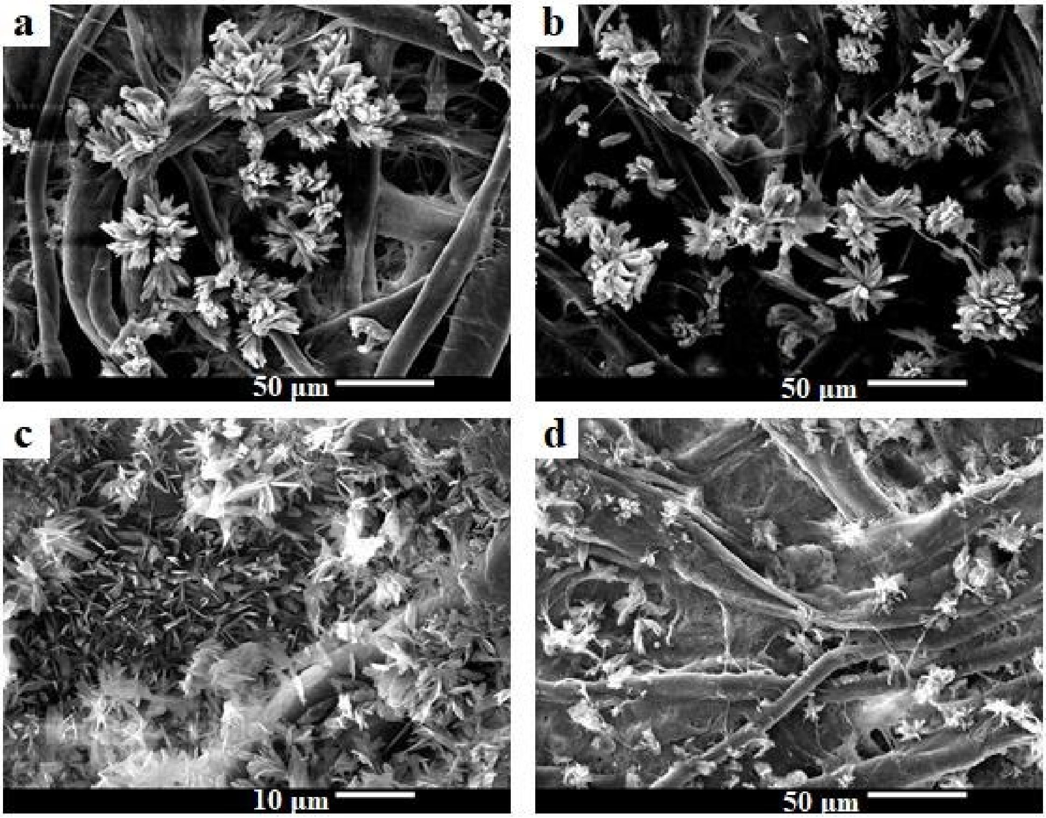

Figure S15 Scanning electron microscopy images of (a) Eu-BDC MOF@CP, (b) Tb-BDC MOF@CP, (c) Tb-BPDC MOF@CP, and (d) Eu-BPDC MOF@CP.

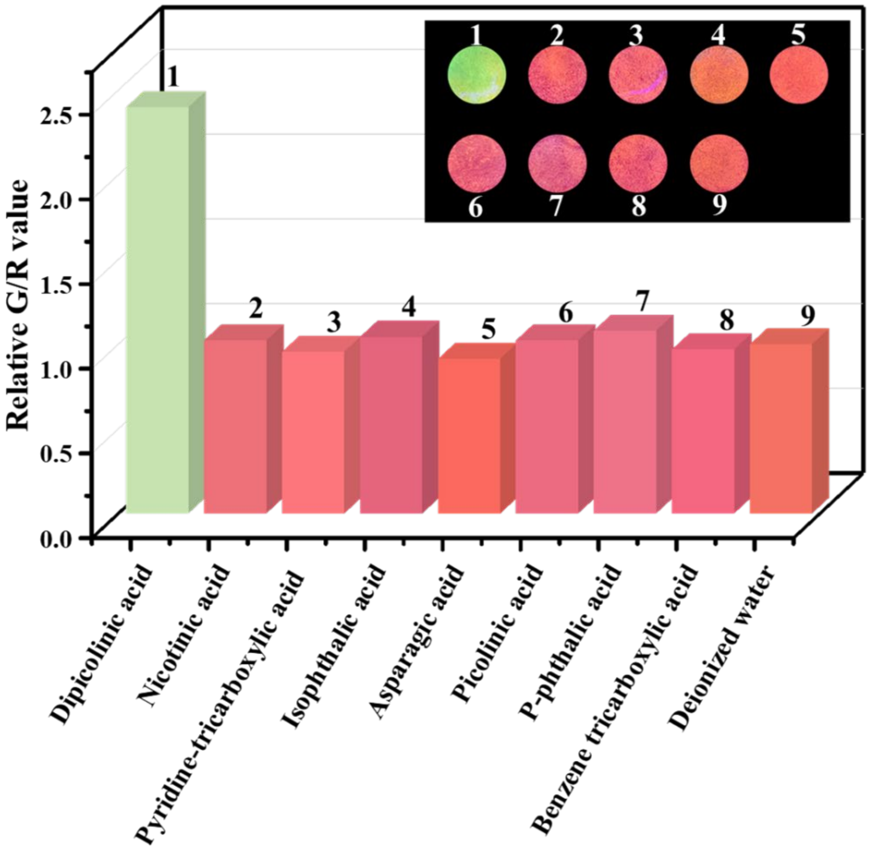

Figure S16 (a) Relative G/R value of $Eu_{0.1}Tb_{0.4}$-MOF@CP upon addition of DPA, (b) Corresponding fluorescence images under 254 nm UV lamp.All substances were tested at a final concentration of 2000 uM in aqueous solution.
